# Approaching optimal entangling collective measurements on quantum computing platforms

**DOI:** 10.1038/s41567-022-01875-7

**Published:** 2023-01-12

**Authors:** Lorcán O. Conlon, Tobias Vogl, Christian D. Marciniak, Ivan Pogorelov, Simon K. Yung, Falk Eilenberger, Dominic W. Berry, Fabiana S. Santana, Rainer Blatt, Thomas Monz, Ping Koy Lam, Syed M. Assad

**Affiliations:** 1 Centre for Quantum Computation and Communication Technology, Department of Quantum Science, Australian National University, Canberra, Australian Capital Territory Australia; 2 Institute of Applied Physics, Abbe Center of Photonics, Friedrich-Schiller University of Jena, Jena, Germany; 3 Cavendish Laboratory, University of Cambridge, Cambridge, UK; 4 Institute for Experimental Physics, Innsbruck, Austria; 5 Fraunhofer Institute for Applied Optics and Precision Engineering IOF, Jena, Germany; 6 Max Planck School of Photonics, Jena, Germany; 7 School of Mathematical and Physical Sciences, Macquarie University, Sydney, New South Wales Australia; 8 Amazon Web Services, Canberra, Australian Capital Territory Australia; 9 Institute for Quantum Optics and Quantum Information, Innsbruck, Austria; 10 Alpine Quantum Technologies (AQT), Innsbruck, Austria; 11 School of Physical and Mathematical Sciences, Nanyang Technological University, Singapore, Republic of Singapore; 12 Institute of Materials Research and Engineering, Agency for Science Technology and Research (A*STAR), Innovis, Singapore

**Keywords:** Quantum metrology, Quantum information, Quantum mechanics, Qubits, Theoretical physics

## Abstract

Entanglement is a fundamental feature of quantum mechanics and holds great promise for enhancing metrology and communications. Much of the focus of quantum metrology so far has been on generating highly entangled quantum states that offer better sensitivity, per resource, than what can be achieved classically. However, to reach the ultimate limits in multi-parameter quantum metrology and quantum information processing tasks, collective measurements, which generate entanglement between multiple copies of the quantum state, are necessary. Here, we experimentally demonstrate theoretically optimal single- and two-copy collective measurements for simultaneously estimating two non-commuting qubit rotations. This allows us to implement quantum-enhanced sensing, for which the metrological gain persists for high levels of decoherence, and to draw fundamental insights about the interpretation of the uncertainty principle. We implement our optimal measurements on superconducting, trapped-ion and photonic systems, providing an indication of how future quantum-enhanced sensing networks may look.

## Abstract (web summary)

Multi-parameter metrology requires collective measurements on more than one copy of the same quantum state. Now, an optimal scheme for the estimation of qubit rotations has been demonstrated on superconducting and trapped-ion platforms.

## Main

Quantum-enhanced single-parameter estimation is an established capability, with non-classical probe states achieving precisions beyond what can be reached by the equivalent classical resources in photonic^1–3^, trapped-ion^4,5^, superconducting^6^ and atomic^7,8^ systems. This has paved the way for quantum enhancements in practical sensing applications, from gravitational wave detection^9^ to biological imaging^10^. For single-parameter estimation, entangled probe states are sufficient to reach the ultimate allowed precisions. However, for multi-parameter estimation, owing to the possible incompatibility of different observables, entangling resources are also required at the measurement stage. The ultimate attainable limits in quantum multi-parameter estimation are set by the Holevo Cramér–Rao bound (Holevo bound)^11,12^. In most practical scenarios, it is not feasible to reach the Holevo bound as this requires a collective measurement on infinitely many copies of the quantum state^13–16^ (see Methods for a rigorous definition of collective measurements). Nevertheless, it is important to develop techniques that will enable the Holevo bound to be approached, given that multi-parameter estimation is fundamentally connected to the uncertainty principle^17^ and has many physically motivated applications, including simultaneously estimating phase and phase diffusion^18,19^, quantum super-resolution^20,21^, estimating the components of a three-dimensional field^22,23^ and tracking chemical processes^24^. Furthermore, as we demonstrate, collective measurements offer an avenue to quantum-enhanced sensing even in the presence of large amounts of decoherence, unlike the use of entangled probe states^25,26^.

To date, collective measurements for quantum multi-parameter metrology have been demonstrated exclusively on optical systems^27–32^. Contemporary approaches to collective measurements on optical systems are limited in their scalability: that is, it is difficult to generalize present approaches to measuring many copies of a quantum state simultaneously. The limited gate set available can also make it harder to implement an arbitrary optimal measurement. Indeed, the collective measurements demonstrated so far have all been restricted to measuring two copies of the quantum state and, while quantum enhancement has been observed, have all failed to reach the ultimate theoretical limits on separable measurements^33,34^. Thus, there is a pressing need for a more versatile and scalable approach to implementing collective measurements.

In this work, we design and implement theoretically optimal collective measurement circuits on superconducting and trapped-ion platforms. The ease with which these devices can be reprogrammed, the universal gate set available and the number of modes across which entanglement can be generated, ensure that they avoid many of the issues that current optical systems suffer from. Using recently developed error mitigation techniques^35^ we estimate qubit rotations about the axes of the Bloch sphere with a greater precision than what is allowed by separable measurements on individual qubits. This approach allows us to investigate several interesting physical phenomena: (1) we demonstrate both optimal single- and two-copy collective measurements reaching the theoretical limits^33,34^. We also implement a three-copy collective measurement as a first step towards surpassing two-copy measurements. However, due to the circuit complexity, this measurement performs worse than single-copy measurements. (2) We investigate the connection between collective measurements and the uncertainty principle. Using two-copy collective measurements, we experimentally violate a metrological bound based on known, but restrictive uncertainty relations^36^. (3) Finally, we compare the metrological performance of quantum processors from different platforms, providing an indication of how future quantum metrology networks may look.

## Theoretical results

**Fig. 1:**
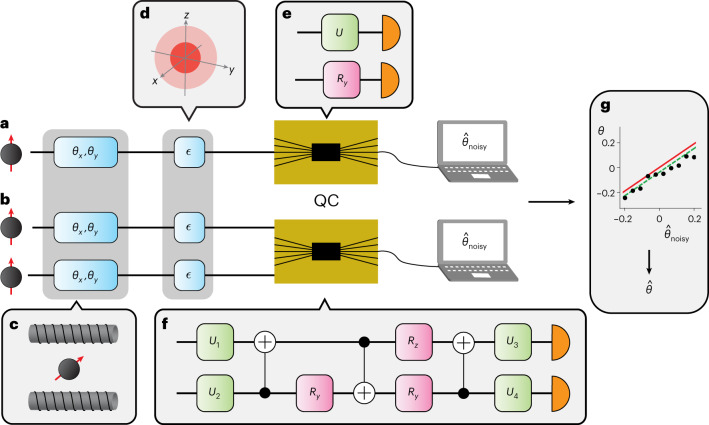
Experimental implementation of optimal collective measurements using quantum computers. **a**,**b**, Probe states are sent to the quantum computers (QC) individually for the single-copy measurement (**a**) and in pairs for the two-copy measurement (**b**). **c**,**d**, The qubit probes experience rotations, *θ*_*x*_ and *θ*_*y*_, about the *x* and *y* axes of the Bloch sphere (**c**) before undergoing decoherence that has the effect of shrinking the Bloch vector (**d**). This rotation can be thought of as being caused by an external magnetic field that we wish to sense. **e**,**f**, The QCs then implement quantum circuits corresponding to the optimal single-copy (**e**) and two-copy (**f**) measurements. Two optimal single-copy circuits are shown, one for estimating *θ*_*x*_ and one for *θ*_*y*_. **g**, Finally, error mitigation is used to improve the accuracy of the estimated angle. We create a model (green line) for how the noisy estimate of *θ*, ${\hat{\theta }}_{{{{\rm{noisy}}}}}$ (black dots), is related to the true value (red line). The model is then used to correct ${\hat{\theta }}_{{{{\rm{noisy}}}}}$ to produce the final estimate $\hat{\theta }$. Sample data from the F-IBM QS1 device downsampled by a factor of three are shown in **g**. Error bars are smaller than the markers.

In this work we implement theoretically optimal quantum circuits saturating the Nagaoka bound^33,34^, which sets an upper limit on the precision attainable with separable measurements. We consider the probe $\left\vert \psi \right\rangle =\left\vert 0\right\rangle$, which experiences small rotations, *θ*_*x*_ and *θ*_*y*_, about the *x* and *y* axes of the Bloch sphere, respectively, before getting decohered (Fig. 1c,d). For small rotations, the state becomes ${\rho }_{1}\approx (1-\epsilon )\left\vert 0\right\rangle \left\langle 0\right\vert +\epsilon /2{\mathbb{1}}$, where *ϵ* is the decoherence strength. Such a noise model is relevant for quantum computing^37^ and communication^38^ among other applications. The Nagaoka bound is given by

1 $${v}_{x}+{v}_{y}\ge {{{{\mathcal{N}}}}}_{1}=\frac{4}{{(1-\epsilon )}^{2}}\ ,$$

where *v*_*x*(*y*)_ is the variance in the estimate of *θ*_*x*(*y*)_. This applies when the probe states are measured one by one (Fig. 1a). We shall refer to measurements of this type as single-copy measurements. The two-copy Nagaoka bound is

2 $${v}_{x}+{v}_{y}\ge {{{{\mathcal{N}}}}}_{2}=\frac{4-2\epsilon +{\epsilon }^{2}}{2{(1-\epsilon )}^{2}}\ ,$$

which applies when we can perform a collective measurement on two copies of the probe, *ρ*_2_ = *ρ*_1_ ⊗ *ρ*_1_, which are entangled during the measurement (Fig. 1b). These measurements are referred to as two*-*copy measurements. Finally, allowing for collective measurements on infinitely many copies of the probe state, the Holevo bound is

3 $${v}_{x}+{v}_{y}\ge {{{\mathcal{H}}}}=\mathop{\lim }\limits_{m\to \infty }m\times {{{{\mathcal{N}}}}}_{m}=\frac{4-2\epsilon }{{(1-\epsilon )}^{2}}\ .$$

The hierarchy between the bounds is, ${{{\mathcal{H}}}}\le 2{{{{\mathcal{N}}}}}_{2}\le {{{{\mathcal{N}}}}}_{1}$, with equality only for *ϵ* = 0 or 1. Detail on the computation of the different bounds is given in Supplementary Note 1.

The Nagaoka bounds, equations (1) and (2), can be saturated by positive operator valued measures (POVMs) in two- and four-dimensional Hilbert spaces, respectively (detailed in Supplementary Note 2). For single-copy measurements, it is possible to measure *θ*_*x*_ and *θ*_*y*_ separately, with two different POVMs, each using half of the total probe states without any loss in precision (Fig. 1e). For the two-copy measurement, this is not possible; both parameters have to be estimated simultaneously to take advantage of the collective measurement. To implement the optimal POVMs, we find a unitary matrix that diagonalizes each POVM in the computational basis. Using standard techniques from quantum computing, we then convert these unitary matrices to quantum circuits^39^, which can be executed experimentally (Fig. 1e,f). We present three- and four-copy POVMs, and the corresponding quantum circuits, which theoretically surpass the two- and three-copy Nagaoka bounds, respectively, in Supplementary Notes 3 and 4.

**Fig. 2:**
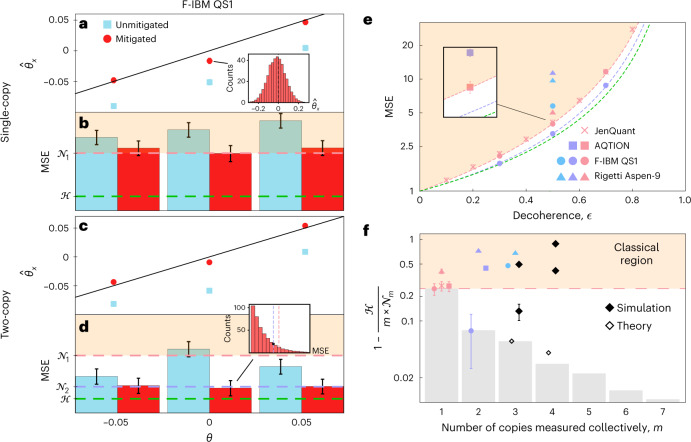
Surpassing single-copy limits through collective measurements. In all figures the dashed pink, purple and green lines correspond to the single-copy Nagaoka, two-copy Nagaoka and Holevo bounds, respectively. The orange-shaded region corresponds to the m.s.e. attainable with separable measurements. m.s.e.s below the dashed green line are forbidden by quantum mechanics. Error bars are obtained using the bootstrapping technique^52^ and correspond to one standard deviation. All experimental points have error bars but some are smaller than the marker size. Each data point corresponds to the average of 400 individual experimental runs, each using 512 shots, as shown in the inset of **a** (see Methods for details). **a**,**c**, Single-copy (**a**) and two-copy (**c**) estimates of *θ*_*x*_, both with and without error mitigation. Results for estimating *θ*_*y*_ are similar (Extended Data Fig. 1). **b**,**d**, The corresponding m.s.e.: single-copy (**b**) and two-copy (**d**). The distribution of m.s.e. values follows the expected chi-squared distribution, shown in the inset of **d**. The black circle in the inset corresponds to the mean m.s.e. value. The results shown in **a**–**d** are for decoherence parameter *ϵ* = 0.5 and are obtained on the F-IBM QS1 device. **e**, Optimal single-, two- and three-copy measurements at different decoherence strengths, *ϵ*. The pink, purple and blue markers correspond to experimental single-, two- and three-copy measurements, respectively. For the superconducting devices, all markers correspond to the precision after using error mitigation. The results of the AQTION trapped-ion processor for *ϵ* = 0.5 are shown in the inset for clarity. **f**, Bars are one minus the ratio of the Holevo bound to the *m*-copy Nagaoka bound, for *m* up to and including 7, calculated theoretically at *ϵ* = 0.5. Experimental points are one minus the ratio of the Holevo bound to the m.s.e. obtained experimentally. Unfilled black diamonds correspond to the precision that our three- and four-copy projective measurements can obtain in theory. The upper and lower filled black diamonds are simulations based on a depolarizing noise model with gate error rates of 5 × 10^−3^ and 1 × 10^−3^, respectively. The legend is the same as in **e**.

We also investigate the asymptotic attainability of the Holevo bound, examining how closely measurements on a finite number of copies of the probe state can approach it. In Fig. 2f, we compute the Nagaoka bound for performing collective measurements on up to seven copies of the probe state simultaneously, corresponding to a 128-dimensional Hilbert space^40^.

## Experimental results

In what follows, we will describe the results of experiments conducted on multiple quantum platforms. The superconducting processors used were the Fraunhofer IBM Q System One (F-IBM QS1) processor, 11 cloud-accessible IBM Q processors and the Rigetti Aspen-9 processor. The trapped-ion processor (AQTION) is described in ref. 41 and the Jena quantum photonic processor (JenQuant) is described in the Methods. We implement the circuits corresponding to the optimal POVMs, shown in Fig. 1e,f, on the superconducting and trapped-ion processors. Additionally, we implement the single-copy measurements on JenQuant. The specific circuit parameters are provided in Supplementary Note 4. The outcomes of each run of a circuit are input to an estimator function to return the estimated values ${\hat{\theta }}_{x}$ and ${\hat{\theta }}_{y}$. This allows the mean squared error (m.s.e.) to be determined.

### Error mitigation for quantum metrology

Our first experiment investigates one possible application of error mitigation to quantum metrology. The details of the error mitigation used are found in Methods, but it is essentially a calibration process based on known angles as shown in Fig. 1g. For this experiment, conducted on the F-IBM QS1 processor, the decoherence parameter is fixed at *ϵ* = 0.5 and we estimate a range of *θ* values. This verifies the unbiasedness of the estimator after error mitigation. Figure 2a,c shows the average estimate of *θ*_*x*_, both before and after error mitigation, with single- and two-copy measurements, respectively. The improvement offered by error mitigation, evident in these figures, is quantified by the m.s.e. in Fig. 2b,d. Error mitigation cannot reduce the m.s.e. below what is theoretically allowed by the Nagaoka bound, but it does enable both the single- and two-copy measurements to reach the corresponding Nagaoka bounds. Crucially, Fig. 2d shows the advantage of the two-copy measurement, achieving a precision beyond what is classically possible over the range of *θ* considered and saturating the two-copy Nagaoka bound. Averaged over the entire range of *θ*, the two-copy measurements show a m.s.e. 19 ± 4% below the theoretical single-copy measurement limit, which is only 6 ± 4% larger than the Holevo bound. In contrast, when restricted to single-copy measurements, the m.s.e. is guaranteed to be at least 33% larger than the Holevo bound. The ability to measure a range of angles is important for practical applications of quantum-enhanced metrology.

### Optimal single-, two- and three-copy measurements

We next fix the rotations to *θ*_*x*_ = *θ*_*y*_ = 0 and demonstrate a quantum enhancement over a range of *ϵ* values. Figure 2e shows the (scaled) m.s.e. attained on different platforms. Using the F-IBM QS1 device, we can demonstrate a clear quantum enhancement across a range of *ϵ* values. The two-copy measurement on the F-IBM QS1 device shows a maximum advantage over the theoretical single-copy limit of 21 ± 4%. In contrast, the Rigetti Aspen-9 superconducting device does not approach the theoretical limits for any of the measurements, likely due to the higher gate and readout error rates. Notably, both JenQuant and the AQTION processor are able to reach the theoretical single-copy measurement limits without any error mitigation. The AQTION processor does not, however, reach the theoretical two-copy limits. The demonstration of quantum-enhanced sensing with highly mixed states showcases that collective measurements may provide metrological gain in real-world sensing applications where decoherence is unavoidable.

In Fig. 2e,f, we show the m.s.e. of our three-copy measurement when implemented on the Rigetti Aspen-9 and F-IBM QS1 processors. In Supplementary Note 6, we present further three-copy results for these and several other devices, all of which failed to reach the theoretical limit and display properties of a bad estimator. These experimental results are in qualitative agreement with simulations of three- and four-copy measurements based on the noise level expected for near-future quantum processors, also shown in Fig. 2f. From Fig. 2f, it is evident that for the problem we have considered, the benefit of three-copy measurements over two-copy measurements is marginal. This raises the question of whether measurements on many copies of a quantum state simultaneously are practically useful. In Supplementary Note 7, we present a similar problem, based on an amplitude damping noise model, where there is a sizeable gap between the two-copy Nagaoka and Holevo bounds, suggesting that collective measurements on many copies may be useful. With continually decreasing error rates, superconducting and trapped-ion devices may bridge this gap and approach the Holevo bound ever more closely. However, as the data from Fig. 2f show, there is a pertinent trade-off between what is gained by measuring more copies of the quantum state and what is lost by the increased experimental complexity.

### Collective measurements and the uncertainty principle

The uncertainty principle is one of the most fundamental features of quantum mechanics^17^. Recently, it has been observed that the original formulations of the uncertainty principle fail to hold in certain scenarios^42,43^, leading to the introduction of ‘universally valid’ uncertainty relations (UVUR) for operators^44–46^. In spite of the name, UVUR assume that measurements are carried out on single copies of the quantum state. This appears to be a natural assumption when considering how the measurement of one quantity disturbs any subsequent measurement of a second quantity. However, the same is not true when considering the precision with which two quantities can be jointly measured. Given this restriction, one might expect that UVUR can be violated through collective measurements.

**Fig. 3:**
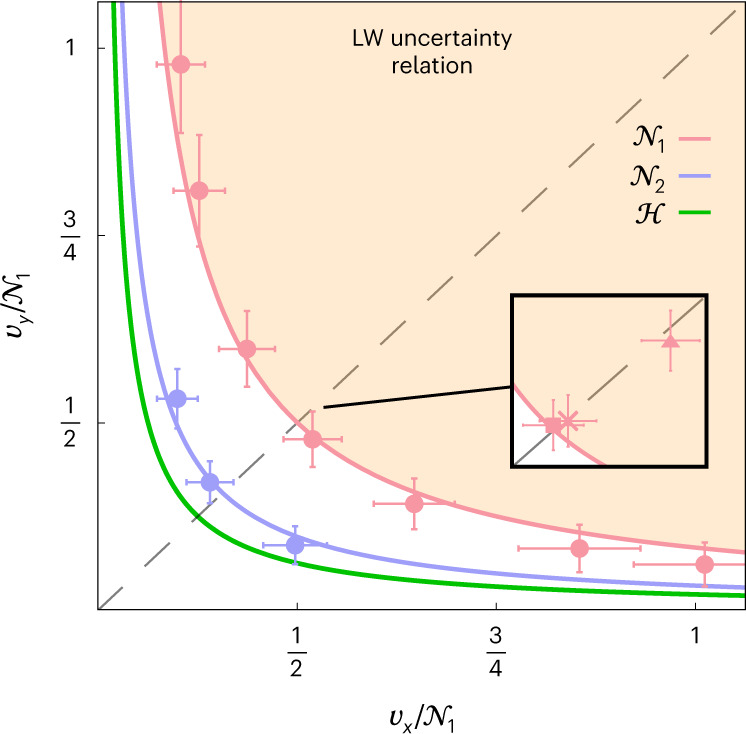
Collective measurements violating the Lu–Wang uncertainty relation. The shaded region shows the measurement variances allowed by the Lu–Wang (LW) uncertainty relation^36^. All experimental points correspond to a decoherence parameter of *ϵ* = 0.5. The dashed grey line shows where the variance in estimating both parameters are equal. The purple and green lines are obtained by calculating the two-copy Nagaoka bound and Holevo bound with different weights attached to each parameter. Solid lines are used for bounds on the allowed values (*v*_*x*_, *v*_*y*_) (as opposed to the sum of variances as in Fig. 2). The data in the main figure correspond to the F-IBM QS1 device. Data from the other processors are shown in the inset. The legend is the same as Fig. 2e. Each data point corresponds to the average of 400 individual experimental runs, each using 512 shots and error bars correspond to one standard deviation obtained by bootstrapping.

Recently, Lu and Wang extended the UVUR to quantum multi-parameter estimation^36^, deriving a metrological bound on how well two parameters can be simultaneously estimated. We shall denote this the Lu–Wang uncertainty relation. For our problem, this bound can be calculated as (Supplementary Note 8):

4 $$\frac{1}{{v}_{x}}+\frac{1}{{v}_{y}}\le {(1-\epsilon )}^{2},$$

which is saturated when *v*_*x*_ = *v*_*y*_ = 2/(1 − *ϵ*)^2^. The variances allowed by equation (4) coincide with our single-copy measurement variances. Indeed, our single-copy measurement variances, shown in pink in Fig. 3, verify the validity of UVUR in this scenario. However, our two-copy measurements implemented on the F-IBM QS1 processor were able to experimentally violate the Lu–Wang uncertainty relation by more than three standard deviations as shown in purple in Fig. 3. The POVMs that give rise to the unbalanced variances are presented in Supplementary Note 9. The observation, both theoretically and experimentally, that UVUR can be surpassed has importance for the manner in which the uncertainty principle is interpreted and indicates that tighter uncertainty relations are required when allowing for any measurement type. In Supplementary Note 10 we relate the violation of the Lu–Wang uncertainty relation to the more common error-disturbance operator uncertainty relations.

### Cross-platform comparison

**Fig. 4:**
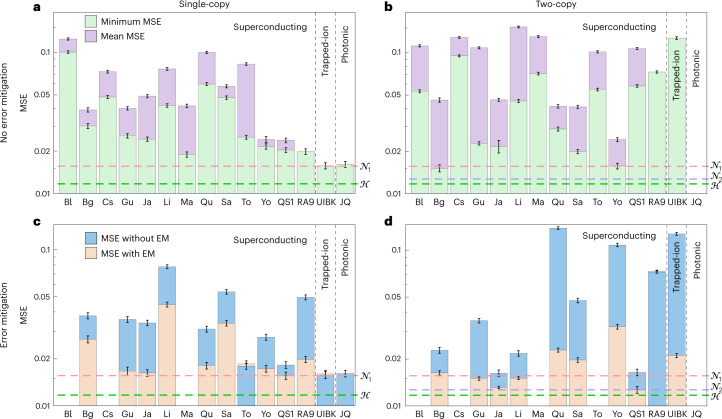
Comparing optimal measurement circuits on different quantum processors. **a**,**b**, The mean m.s.e. and minimum m.s.e. across all qubits with different quantum processors for the single- (**a**) and two-copy (**b**) measurements, respectively. No error mitigation is used in these figures. Each m.s.e. is averaged over 600 experimental runs, corresponding to five different angles, each using 512 shots. **c**,**d**, The m.s.e. with and without error mitigation (EM) for the single- and two-copy measurements, respectively. In all but four cases error mitigation is beneficial. The data in **c** and **d** correspond to the average of 400 individual experimental runs, each using 512 shots. For all figures, error bars correspond to one standard deviation obtained by bootstrapping. The different IBM Q processors tested are Belem (Bl), Bogota (Bg), Casablanca (Cs), Guadalupe (Gu), Jakarta (Ja), Lima (Li), Manhattan (Ma), Quito (Qu), Santiago (Sa), Toronto (To), Yorktown (Yo) and the F-IBM QS1 device (QS1). Also shown is the Rigetti Aspen-9 superconducting device (RA9), JenQuant (JQ) and the AQTION trapped-ion device (UIBK). For the Rigetti Aspen-9 device, only one qubit or pair of qubits was tested, hence the mean m.s.e. and minimum m.s.e. are equal. For the AQTION device, the mean m.s.e. and minimum m.s.e. are equal as only one ion or pair of ions was loaded into the trap. Empty spaces correspond to processors where a particular experiment could not be carried out.

Our final experiment compares the performance of different platforms for estimating qubit rotations. This provides an indication of what resources may be used in a future quantum metrology network. For superconducting devices, we first perform simultaneous qubit rotation estimation using all non-neighbouring (pairs of) qubits, to minimize cross-talk between qubits. The mean m.s.e. and minimum m.s.e. across all qubits is shown in Fig. 4a,b for each device tested. Each m.s.e. is averaged over estimating five angles in the range *θ* = −0.01 to 0.01, repeated 120 times for each angle. For the trapped-ion and photonic devices only one photon, ion or pair of ions was used, hence only the mean m.s.e. is shown. We then repeat the experiment using only the best performing qubit(s), now applying error mitigation as shown in Fig. 4c,d. The benefits of error mitigation are most pronounced for the F-IBM QS1 processor as we had unrestricted access to this device. Having restricted access to a device means each experiment takes longer, hence the model for the device provided by error mitigation is likely to be less accurate by the end of the experiment.

## Discussion

Superconducting and trapped-ion devices are natural platforms for attaining the maximal advantage of quantum metrology and quantum information tasks through collective measurements. By implementing collective measurements on pairs of quantum states, we have been able to perform quantum multi-parameter estimation with a precision that cannot be reached classically using the same resources. There are many scenarios where this work may prove beneficial, particularly when there is an intrinsic restriction on resources. One can envision an optical system connected to a quantum processor through optical-to-microwave converters^47^. With only a limited number of qubits, such a device could greatly enhance biomedical imaging or quantum communications, meaning these advantages may be leveraged with near-future technology. Furthermore, collective measurements can be beneficial for quantum tomography^48^, entanglement distillation for quantum communication^49^ and quantum illumination^50^.

This work opens up a number of avenues for future investigation: a natural extension to using error mitigation for quantum metrology is error correction^51^. With the aid of the techniques presented here, it may be possible to demonstrate multi-parameter metrology that fully utilizes quantum resources; benefiting from both entangled probe states and collective measurements. By simplifying our three-copy measurement circuit, the theoretical limits may be approachable with the present generation of quantum processors. It would also be pertinent to study further how gate error rates and circuit complexity need to scale to successfully implement many-copy collective measurements. Investigating further the connection between collective measurements and the uncertainty principle may reveal important aspects of fundamental physics and could lead to the development of tighter uncertainty relations that hold true for any measurement type. Finally, the ideal extension of our work is to demonstrate optimal collective measurements in a practical setting. We anticipate that our work brings this closer.

## Methods

### Collective measurements

Here we clarify our use of terminology regarding ‘entangling’ and ‘collective’ measurements. We stick to the definitions used in refs. 27–32, where a collective measurement is a measurement that acts on multiple copies of the quantum state simultaneously. An *m*-copy collective measurement thus simultaneously measures *m* copies of the same state, whereas a ‘single-copy’ measurement, or ‘separable’ measurement, measures the quantum states individually. The quantum states themselves may consist of an arbitrary number of possibly entangled modes. When we refer to ‘entangling’ measurements we mean measurements capable of creating entanglement between multiple copies of the quantum state, or alternatively, in an entangled multi-copy basis.

There are many similar concepts, which may be confused with our definition of a collective measurement. For example, in ref. 53 a quantum state with 26 entangled modes (ions) was used. In our terminology, measuring the 26 ions simultaneously is a separable measurement, because only a single copy of the quantum state was used and consequently no entanglement between copies was possible. However, in principle the (0,2) and (1,2) schemes in ref. 53 could be used for implementing collective measurements in the sense of our definition. Similarly, ref. 54 refers to collective measurements as measurements of ensemble quantities of atoms, wholly unrelated to our terminology. In ref. 55 multi-copy discrimination of two quantum states is demonstrated. However, this multi-copy discrimination uses separable measurements, the multi-copy part referring to the fact that multiple (separable) measurement outcomes are used in making a final decision. Finally, refs. 56, 57 examine multi-copy metrology. Again, in this work, the term multi-copy carries a different meaning compared to our work, as only single-parameter estimation was considered.

### Photonic experiment

The Jena quantum photonic processor (JenQuant) is based on a single photon emitting colour centre in the two-dimensional material hexagonal boron nitride (hBN). The crystal defect introduces an effective two-level system into the bandgap that is excited optically. The emitter is fabricated by treating a multilayer hBN crystal with an oxygen plasma and subsequent rapid thermal annealing^58^. A suitable quantum emitter was then coupled to a hemispherical microcavity^59^. The resonator enhances the emission via the Purcell effect and suppresses noise to reduce the multi-photon probability below 0.6% at room temperature^60^. The spectrum is tunable by adjusting the resonator length within the free space emission linewidth of 5.76 nm (full-width at half-maximum) around 565 nm and has a linewidth of 0.2 nm (ref. 59).

We encode the logical qubits in the polarization of the photons and choose $\left\vert {{{{H/V}}}}\right\rangle$ as the computational basis states $\left\vert 0/1\right\rangle$. The input states $\left\vert 0\right\rangle$, $\left\vert 1\right\rangle$, and $\left\vert {\psi }_{\theta }\right\rangle$ are set by motorized polarization optics (a half-wave plate, polarizer and a quarter-wave plate (QWP)). The polarizer ensures a high polarization extinction ratio of >10^5^:1. The single-copy POVMs are implemented by the combination of motorized QWP, half-wave plate and QWP, which can perform any arbitrary unitary rotation. In Supplementary Note 4 we show the decomposition of the optimal single-copy POVMs into wave plate rotations. Finally, a polarizing beam splitter projects onto the computational basis and the photons are detected by two single photon detectors in both arms. JenQuant is thereby a fully universal single qubit quantum computer. Performing multi-qubit operations requires an entangling gate, such as a controlled NOT gate, which would require indistinguishable single photons. This in turn can be achieved by a narrower resonator linewidth <124 MHz to reach a Hong-Ou-Mandel contrast >90% (ref. 59). Note that JenQuant does not require any error mitigation, partly due to the long-term stability of the system.

### Superconducting experiments

The F-IBM QS1 device used is based in Ehningen. It uses an IBM Quantum Falcon processor and has 27 qubits. As with all IBM Quantum devices, the qubits are transmons. The frequency of the transmons are around 5 GHz (refs. 61).

### Error mitigation

Before running each experiment for estimating the unknown angles *θ*_*x*_ and *θ*_*y*_, we implement Clifford data regression error mitigation^35^. This involves constructing a model for how a noisy expectation value predicted by a quantum processor is related to the true expectation value. In general complex models can be used, however, for quantum metrology, it is essential that the chosen model does not bias the estimator. We are therefore required to use a simple model of the form ${\hat{\theta }}_{x(y)}={\hat{\theta }}_{{{{\rm{noisy,}}}}x(y)}+{c}_{x(y)}$, where ${\hat{\theta }}_{{{{\rm{noisy,}}}}x(y)}$ is the unmitigated *θ*_*x*(*y*)_ value predicted by the quantum processor and *c*_*x*(*y*)_ is a constant. Detail on other possible models that were considered, but found to bias the estimator, is provided in Supplementary Note 5. We use 30 known *θ* values in the range *θ* ∈ [−0.2, 0.2] rad to determine a value for the model *c*_*x*(*y*)_. An example of the model fitting is shown in Fig. 1g for the F-IBM QS1 quantum processor. This model is then used to estimate some unknown angle *θ* = *θ*_*x*_ = *θ*_*y*_. Unless otherwise specified in the main text, the model is recalibrated after every 40 predictions of the unknown angle and the process is repeated to estimate each unknown angle 400 times. Our figure of merit is taken to be the average m.s.e. over all 400 runs.

5 $${{{\rm{m.s.e.}}}}=\frac{1}{400}\mathop{\sum }\limits_{i=1}^{400}({({\theta }_{x}-{\hat{\theta }}_{x,i})}^{2}+{({\theta }_{y}-{\hat{\theta }}_{y,i})}^{2}),$$

where ${\hat{\theta }}_{x(y),i}$ is the *i*th estimate of *θ*_*x*(*y*)_. To obtain each of the 400 estimates, we average the results of 512 repetitions of the experiment for each of the single-copy circuits and for the two-copy circuit. For the three-copy circuit, we average the results of 341 repetitions of the experiment to ensure equal resources are used in each experiment.

For the two-copy measurements in Fig. 3 with *v*_*x*_ ≠ *v*_*y*_, a slightly different error mitigation process was used. At the time these particular data were being taken, it was not possible to recalibrate in between estimating the unknown angle. Hence, the calibration step was only performed once, immediately before estimating the unknown angle. To increase the utility of the error mitigation in this case, we used 30 known angles in the range *θ* ∈ [−0.05, 0.05] rad.

## Online content

Any methods, additional references, Nature Portfolio reporting summaries, source data, extended data, supplementary information, acknowledgements, peer review information; details of author contributions and competing interests; and statements of data and code availability are available at 10.1038/s41567-022-01875-7.

## Supplementary information

Supplementary Information Supplementary Notes 1–12, Figs. 1–8 and Tables 1 and 2.

## Figures and Tables

**Extended Data Fig. 1:**
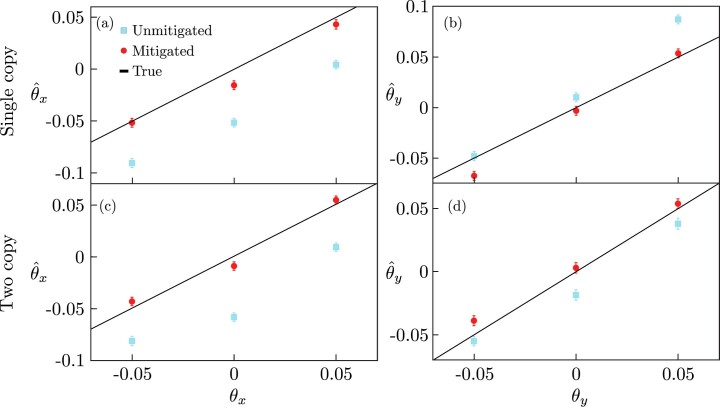
Effect of error mitigation on estimation performance. Figs (a) to (d) show the estimated values of *θ*, averaged over all 400 runs, before (blue squares) and after (red circles) applying error mitigation. Figs (a) and (b) ((c) and (d)) correspond to estimating *θ*_*x*_ and *θ*_*y*_ respectively with the optimal single(two)-copy measurement. Error bars are obtained using the bootstrapping technique and correspond to one standard deviation. All results shown are for decoherence parameter *ϵ* = 0.5 and are obtained on the F-IBM QS1 device. Each data point corresponds to the average of 400 individual experimental runs, each using 512 shots.

## Data Availability

All data are available at the following Github repository: https://github.com/LorcanConlon/Approaching-optimal-entangling-collective-measurements.

## Code availability

All codes are available at the following Github repository: https://github.com/LorcanConlon/Approaching-optimal-entangling-collective-measurements.
