# Supplementary material for: Approaching optimal entangling collective measurements on quantum computing platforms
Source: Nat Phys. 2023 Jan 12;19(3):351–7. doi: 10.1038/s41567-022-01875-7 (PMC10020085; doi:10.1038/s41567-022-01875-7)
Supplement: Supplementary file 1 — Supplementary Notes 1–12, Figs. 1–8 and Tables 1 and 2. [file 41567_2022_1875_MOESM1_ESM.pdf]

nature physics

Article

https://doi.org/10.1038/s41567-022-01875-7

Approaching optimal entangling collective measurements on quantum computing platforms

In the format provided by the authors and unedited

TABLE OF CONTENTS

Supplementary Note 1 .	Computation of the Holevo and Nagaoka bounds	2
Supplementary Note 2 .	POVMs saturating the Nagaoka bounds	3
Supplementary Note 3 .	Optimised three- and four-copy projective measurements surpassing the preceding Nagaoka bound	5
Supplementary Note 4 .	Experimental implementation of optimal POVMs	6
	1. Single- and two-copy POVM implementation	6
	2. State preparation	7
	3. Three- and four-copy POVM implementation and noisy simulation	9
Supplementary Note 5 .	Effect of error mitigation on the bias of an estimator	10
Supplementary Note 6 .	Further three-copy measurement results	11
Supplementary Note 7 .	Problem where collective measurements on many copies of the probe state are necessary	12
Supplementary Note 8 .	Computing Lu and Wang's metrological bound based on Heisenberg's uncertainty principle	14
Supplementary Note 9 .	POVMs violating the LW uncertainty relation with unbalanced variances	15
Supplementary Note 10 .	Measurement surpassing universally valid uncertainty relations for operators	16
Supplementary Note 11 .	Adjusting Lu and Wang's bound to allow for collective measurements	18
Supplementary Note 12 .	Probability simplex	19

Supplementary Note 1 . Computation of the Holevo and Nagaoka bounds

In this Supplementary Note we describe the computation of the single-copy Nagaoka, two-copy Nagaoka and Holevo bounds. For the qubit rotation estimation problem discussed in this work we consider the state $|0\rangle$ subject to rotations θ_x and θ_y about the x and y axes of the Bloch sphere respectively. The rotation operators are given by

$$R_x(\theta) = \begin{pmatrix} \cos(\frac{\theta}{2}) & -\mathrm{i}\,\sin(\frac{\theta}{2}) \\ -\mathrm{i}\,\sin(\frac{\theta}{2}) & \cos(\frac{\theta}{2}) \end{pmatrix} \qquad \text{and} \qquad R_y(\theta) = \begin{pmatrix} \cos(\frac{\theta}{2}) & -\sin(\frac{\theta}{2}) \\ \sin(\frac{\theta}{2}) & \cos(\frac{\theta}{2}) \end{pmatrix} \, . \tag{1}$$

After the rotations, the probe is subject to the decoherence channel. Assuming the rotations are small, the density matrix and its derivatives are given by

$$\rho = \begin{pmatrix} 1-\frac{\epsilon}{2} & 0 \\ 0 & \frac{\epsilon}{2} \end{pmatrix} \qquad \text{and} \qquad \partial_x\rho = \begin{pmatrix} 0 & \frac{\mathrm{i}}{2}(1-\epsilon) \\ \frac{-\mathrm{i}}{2}(1-\epsilon) & 0 \end{pmatrix} \qquad \text{and} \qquad \partial_y\rho = \begin{pmatrix} 0 & \frac{(1-\epsilon)}{2} \\ \frac{(1-\epsilon)}{2} & 0 \end{pmatrix} \, , \tag{2}$$

where ϵ is the decoherence strength and ∂_i represents the partial derivative with respect to the parameter θ_i. The parameters θ_x and θ_y can be estimated using a positive operator valued measure (POVM). A POVM is described by a set of non-negative operators, $\{\Pi_l\}$, which sum to the identity, $\sum_l \Pi_l = \mathbb{I}$. Each measurement outcome occurs with a certain state-dependent probability, $p_l = \mathrm{tr}[\rho_\theta \Pi_l]$. The series of measurement results are used to construct unbiased estimators, $\hat{\theta}_x$ and $\hat{\theta}_y$, for the parameters of interest, θ_x and θ_y. Our goal is to minimise the mean squared error (MSE) between the true θ and the estimated value $\hat{\theta}$. The MSE matrix is given by

$$[V(\hat{\theta})]_{jk} = \sum_l (\hat{\theta}_j(l) - \theta_j)(\hat{\theta}_k(l) - \theta_k) p_l \, , \tag{3}$$

where the sum is over all possible measurement outcomes. The Holevo and Nagaoka bounds are lower bounds for the trace of the MSE matrix when using collective and separable measurements respectively.

The Holevo bound is obtained by solving the following non-trivial minimisation problem [1, 2]

$$\mathrm{Tr}[V] \geq \mathcal{H} := \min_X \mathrm{Tr}[Z_\theta[X]] + \mathrm{TrAbs}[\mathrm{Im}\, Z_\theta[X]] \, , \tag{4}$$

where $Z_\theta[X]_{jk} := \mathrm{Tr}[\rho X_j X_k]$, $\mathrm{Im}Z_\theta[X]$ represents the imaginary part of $Z_\theta[X]$, and $\mathrm{TrAbs}[\mathrm{Im}Z_\theta[X]]$ is the sum of the absolute values of the eigenvalues of the matrix $\mathrm{Im}Z_\theta[X]$. The minimisation is over the Hermitian matrices X, subject to the unbiased conditions

$$\begin{aligned} \mathrm{Tr}[\rho X_i] &= 0 \\ \mathrm{Tr}[\partial_i \rho X_j] &= \delta_{ij} \, . \end{aligned} \tag{5}$$

The Holevo bound applies when we allow for collective measurements on infinitely many copies of the probe state. When restricted to separable measurements, the Nagaoka bound provides an upper limit on the attainable precision. For estimating two parameters, the Nagaoka bound is given by [3, 4]

$$\mathrm{Tr}[V] \geq \mathcal{N} := \min_X \mathrm{Tr}[Z_\theta[X]] + \mathrm{TrAbs}[\rho[X_1, X_2]] \, , \tag{6}$$

where $[A, B] = AB - BA$ and the Hermitian matrices X are subject to the same unbiased conditions as before.

The following Hermitian matrices simultaneously solve the optimisation problem for the Holevo bound and the single-copy Nagaoka bound

$$X_x = \begin{pmatrix} 0 & \frac{\mathrm{i}}{1-\epsilon} \\ \frac{-\mathrm{i}}{1-\epsilon} & 0 \end{pmatrix} \qquad \text{and} \qquad X_y = \begin{pmatrix} 0 & \frac{1}{1-\epsilon} \\ \frac{1}{1-\epsilon} & 0 \end{pmatrix} \, . \tag{7}$$

By substituting into Eq. (4) it is easily verified that these matrices give a Holevo bound of

$$v_x + v_y \geq \mathcal{H} = \frac{4-2\epsilon}{(1-\epsilon)^2} \;, \tag{8}$$

where $v_{x(y)}$ is the variance in the estimate of $\theta_{x(y)}$. Similarly, by substituting into Eq. (6), the single-copy Nagaoka bound is given by

$$v_x + v_y \geq \mathcal{N}_1 = \frac{4}{(1-\epsilon)^2} \;. \tag{9}$$

For computing the two-copy Nagaoka bound it is no longer sufficient to consider the state and derivatives in Eq. (2). To find the attainable precision when performing collective measurements on two copies of the quantum state simultaneously, we make the transformation

$$\rho \rightarrow \rho \otimes \rho, \qquad \partial_x\rho \rightarrow \partial_x\rho \otimes \rho + \rho \otimes \partial_x\rho \qquad \text{and} \qquad \partial_y\rho \rightarrow \partial_y\rho \otimes \rho + \rho \otimes \partial_y\rho \;. \tag{10}$$

The Hermitian matrices which solve the two-copy Nagaoka bound are given by

$$X_x = \begin{pmatrix} 0 & \frac{\mathrm{i}}{2(1-\epsilon)} & \frac{\mathrm{i}}{2(1-\epsilon)} & \frac{1-\mathrm{i}}{2(1-\epsilon)} \\ \frac{-\mathrm{i}}{2(1-\epsilon)} & 0 & 0 & \frac{\mathrm{i}}{2(1-\epsilon)} \\ \frac{-\mathrm{i}}{2(1-\epsilon)} & 0 & 0 & \frac{\mathrm{i}}{2(1-\epsilon)} \\ \frac{1+\mathrm{i}}{2(1-\epsilon)} & \frac{-\mathrm{i}}{2(1-\epsilon)} & \frac{-\mathrm{i}}{2(1-\epsilon)} & 0 \end{pmatrix} \quad \text{and} \quad X_y = \begin{pmatrix} 0 & \frac{1}{2(1-\epsilon)} & \frac{1}{2(1-\epsilon)} & \frac{1+\mathrm{i}}{2(1-\epsilon)} \\ \frac{1}{2(1-\epsilon)} & 0 & 0 & \frac{1}{2(1-\epsilon)} \\ \frac{1}{2(1-\epsilon)} & 0 & 0 & \frac{1}{2(1-\epsilon)} \\ \frac{1-\mathrm{i}}{2(1-\epsilon)} & \frac{1}{2(1-\epsilon)} & \frac{1}{2(1-\epsilon)} & 0 \end{pmatrix} \;. \tag{11}$$

We note that these solutions are not unique and multiple optimal solutions were found for the two-copy Nagaoka bound. This solution gives the two-copy Nagaoka bound as

$$v_x + v_y \geq \mathcal{N}_2 = \frac{4-2\epsilon+\epsilon^2}{2(1-\epsilon)^2} \;. \tag{12}$$

As both the Holevo and Nagaoka bounds can be computed efficiently [5, 6] we can be sure our solutions are correct.

Supplementary Note 2 . POVMs saturating the Nagaoka bounds

In this Supplementary Note we present measurement strategies, i.e. POVMs and estimator functions, which saturate the single- and two-copy Nagaoka bounds. A POVM which saturates the single-copy Nagaoka bound is given by

$$\begin{aligned} \Pi_1 &= \frac{1}{4}(-\mathrm{i}\,|0\rangle + |1\rangle)(\mathrm{i}\,\langle 0| + \langle 1|) \;, \\ \Pi_2 &= \frac{1}{4}(\mathrm{i}\,|0\rangle + |1\rangle)(-\mathrm{i}\,\langle 0| + \langle 1|) \;, \\ \Pi_3 &= \frac{1}{4}(|0\rangle + |1\rangle)(\langle 0| + \langle 1|) \;, \\ \Pi_4 &= \frac{1}{4}(-\,|0\rangle + |1\rangle)(-\,\langle 0| + \langle 1|) \;. \end{aligned} \tag{13}$$

The probability of each of the four outcomes is $\frac{1}{4}$. By attaching an estimator coefficient $\xi_{j,k}$ to each measurement outcome, it is possible to construct unbiased estimators for the parameters we want to sense, $\hat{\theta}_j = \sum_k p_k \xi_{j,k}$, where p_k is the probability of the kth POVM outcome occuring. From this particular POVM, we can construct unbiased estimators using the following estimator coefficients $\xi_{x,2} = \xi_{y,3} = -\xi_{x,1} = -\xi_{y,4} = 2/(1-\epsilon)$ and $\xi_{i,j} = 0$ for all other i and j. These estimators then give individual variances of

$$v_x = \frac{1}{4}(\xi_{x,1}^2 + \xi_{x,2}^2) \;, \tag{14}$$

$$v_y = \frac{1}{4}(\xi_{y,3}^2 + \xi_{y,4}^2) \;, \tag{15}$$

which gives a total variance of $v_x + v_y = 4/(1-\epsilon)^2$ coinciding with the single-copy Nagaoka bound $\mathcal{N}_1$, Eq. (9). Alternatively, from the POVM, it is possible to compute the classical Fisher information without using the estimator coefficients.

This measurement strategy is theoretically optimal when measuring probe states individually. However, it requires four measurement outcomes, meaning it is necessary to use an ancilla qubit to implement this POVM. In this case Naimark's theorem can be used to convert the POVM to a projective measurement in a higher dimensional Hilbert space [7], but this comes at the cost of increasing experimental complexity. This can be avoided by noting that this POVM is equivalent to measuring σ_x half of the time and σ_y half of the time, where σ_x and σ_y are the usual Pauli matrices. Thus we can simply split our measurement in two. We measure θ_x with half of the probe states using the following POVM

$$\begin{aligned}\Pi_{x1} &= \frac{1}{2}(-\mathrm{i}\,|0\rangle + |1\rangle)(\mathrm{i}\,\langle 0| + \langle 1|)\ ,\\ \Pi_{x2} &= \frac{1}{2}(\mathrm{i}\,|0\rangle + |1\rangle)(-\mathrm{i}\,\langle 0| + \langle 1|)\ ,\end{aligned} \tag{16}$$

and θ_y with the remaining probe states using

$$\begin{aligned}\Pi_{y1} &= \frac{1}{2}(|0\rangle + |1\rangle)(\langle 0| + \langle 1|)\ ,\\ \Pi_{y2} &= \frac{1}{2}(-\,|0\rangle + |1\rangle)(-\,\langle 0| + \langle 1|)\ .\end{aligned} \tag{17}$$

The new estimator coefficients are reduced by a factor of two $\xi^*_{x,2} = \xi^*_{y,1} = -\xi^*_{x,1} = -\xi^*_{y,2} = 1/(1-\epsilon)$, the probability of each outcome is now $\frac{1}{2}$ and each estimate uses half as many resources. Therefore the variances in estimating θ_x and θ_y are given by

$$v_x = 2(\frac{1}{2})((\xi^*_{x,1})^2 + (\xi^*_{x,2})^2) = \frac{1}{4}(\xi^2_{x,1} + \xi^2_{x,2})\ , \tag{18}$$

$$v_y = 2(\frac{1}{2})((\xi^*_{y,1})^2 + (\xi^*_{y,2})^2) = \frac{1}{4}(\xi^2_{y,3} + \xi^2_{y,4})\ , \tag{19}$$

coinciding with the original measurement, Eq. (13).

For the two-copy measurement we write the POVM as $\Pi_j = |\psi_j\rangle\langle\psi_j|$ where

$$|\psi_1\rangle = \begin{pmatrix}\frac{1}{\sqrt{3}}\\ \frac{-\exp(\mathrm{i}\pi/4)}{\sqrt{6}}\\ \frac{-\exp(\mathrm{i}\pi/4)}{\sqrt{6}}\\ \frac{\exp(\mathrm{i}\pi/2)}{\sqrt{3}}\end{pmatrix},\qquad |\psi_2\rangle = \begin{pmatrix}\frac{1}{\sqrt{3}}\\ \frac{\exp(-\mathrm{i}\pi/12)}{\sqrt{6}}\\ \frac{\exp(-\mathrm{i}\pi/12)}{\sqrt{6}}\\ \frac{\exp(-\mathrm{i}\pi/6)}{\sqrt{3}}\end{pmatrix},\qquad |\psi_3\rangle = \begin{pmatrix}\frac{1}{\sqrt{3}}\\ \frac{\exp(\mathrm{i}7\pi/12)}{\sqrt{6}}\\ \frac{\exp(\mathrm{i}7\pi/12)}{\sqrt{6}}\\ \frac{\exp(-\mathrm{i}5\pi/6)}{\sqrt{3}}\end{pmatrix},\qquad |\psi_4\rangle = \begin{pmatrix}0\\ \frac{1}{\sqrt{2}}\\ \frac{-1}{\sqrt{2}}\\ 0\end{pmatrix}. \tag{20}$$

The first three POVM outcomes occur with probability $(4+\epsilon(\epsilon-2))/12$ and the fourth outcome occurs with probability $\epsilon(2-\epsilon)/4$. We use the following estimator coefficients

$$\xi_{x,1} = \frac{1}{1-\epsilon},\qquad \xi_{x,2} = \frac{\sqrt{3}-1}{2(1-\epsilon)},\qquad \xi_{x,3} = \frac{-\sqrt{3}-1}{2(1-\epsilon)}\qquad \text{and}\qquad \xi_{x,4} = 0 \tag{21}$$

$$\xi_{y,1} = \frac{-1}{1-\epsilon},\qquad \xi_{y,2} = \frac{\sqrt{3}+1}{2(1-\epsilon)},\qquad \xi_{y,3} = \frac{1-\sqrt{3}}{2(1-\epsilon)}\qquad \text{and}\qquad \xi_{y,4} = 0\ . \tag{22}$$

The individual variances are then given by

$$v_x = \frac{1}{4} + \frac{3}{4(1-\epsilon)^2}\ , \tag{23}$$

$$v_y = \frac{1}{4} + \frac{3}{4(1-\epsilon)^2}\ , \tag{24}$$

and the sum coincides with the two-copy Nagaoka bound, Eq. (12). A geometrical interpretation of this POVM is provided in Supplementary Note 12 .

Supplementary Note 3 . Optimised three- and four-copy projective measurements surpassing the preceding Nagaoka bound

In this Supplementary Note we present the details of the three- and four-copy measurements mentioned in the main text, which surpass the two- and three-copy Nagaoka bounds respectively. A three-copy POVM and estimator function which surpass the two-copy Nagaoka bound were found numerically for $\epsilon = 0.5$. For this particular decoherence strength, the Holevo bound on the variance when measuring θ_x and θ_y, per qubit, is 12 rad^2. The single-, two- and three-copy Nagaoka bounds on the variance are 16 rad^2, 13 rad^2 and 12.716 rad^2 respectively. Our specific three-copy measurement is theoretically able to attain a variance of 12.719, almost the same as the three-copy Nagaoka bound. We use the following 8 POVM elements to obtain this variance.

$$
\left|\psi_1^{3c}\right\rangle = \begin{pmatrix} 0.4993+0.0260\mathrm{i} \\ -0.2887+0.0006\mathrm{i} \\ -0.2887+0.0006\mathrm{i} \\ 0.2882-0.0162\mathrm{i} \\ -0.2887+0.0006\mathrm{i} \\ 0.2882-0.0162\mathrm{i} \\ 0.2882-0.0162\mathrm{i} \\ -0.4970+0.0551\mathrm{i} \end{pmatrix}, \left|\psi_2^{3c}\right\rangle = \begin{pmatrix} 0.4968-0.0563\mathrm{i} \\ 0.2847-0.0480\mathrm{i} \\ 0.2847-0.0480\mathrm{i} \\ 0.2816-0.0633\mathrm{i} \\ 0.2847-0.0480\mathrm{i} \\ 0.2816-0.0633\mathrm{i} \\ 0.2816-0.0633\mathrm{i} \\ 0.4812-0.1359\mathrm{i} \end{pmatrix}, \left|\psi_3^{3c}\right\rangle = \begin{pmatrix} 0.4086-0.2881\mathrm{i} \\ 0.1789+0.2266\mathrm{i} \\ 0.1789+0.2266\mathrm{i} \\ -0.2166+0.1909\mathrm{i} \\ 0.1789+0.2266\mathrm{i} \\ -0.2166+0.1909\mathrm{i} \\ -0.2166+0.1909\mathrm{i} \\ -0.3504-0.3566\mathrm{i} \end{pmatrix},
$$

$$
\left|\psi_4^{3c}\right\rangle = \begin{pmatrix} 0 \\ -0.0076-0.0237\mathrm{i} \\ -0.4459-0.2057\mathrm{i} \\ -0.4754+0.1796\mathrm{i} \\ 0.4535+0.2294\mathrm{i} \\ 0.4528-0.1900\mathrm{i} \\ 0.0226+0.0104\mathrm{i} \\ 0 \end{pmatrix}, \left|\psi_5^{3c}\right\rangle = \begin{pmatrix} 0 \\ 0.0015-0.0248\mathrm{i} \\ -0.3411-0.3532\mathrm{i} \\ 0.5082+0.0049\mathrm{i} \\ 0.3396+0.3781\mathrm{i} \\ -0.4909+0.0130\mathrm{i} \\ -0.0173-0.0179\mathrm{i} \\ 0 \end{pmatrix}, \left|\psi_6^{3c}\right\rangle = \begin{pmatrix} 0 \\ -0.4038+0.4119\mathrm{i} \\ 0.2011-0.2275\mathrm{i} \\ 0.2740+0.0008\mathrm{i} \\ 0.2026-0.1844\mathrm{i} \\ 0.3020-0.0320\mathrm{i} \\ -0.5760+0.0313\mathrm{i} \\ 0 \end{pmatrix}, \tag{25}
$$

$$
\left|\psi_7^{3c}\right\rangle = \begin{pmatrix} 0 \\ 0.5637+0.1225\mathrm{i} \\ -0.2997-0.0491\mathrm{i} \\ 0.1452+0.2323\mathrm{i} \\ -0.2640-0.0734\mathrm{i} \\ 0.1879+0.2386\mathrm{i} \\ -0.3331-0.4709\mathrm{i} \\ 0 \end{pmatrix}, \left|\psi_8^{3c}\right\rangle = \begin{pmatrix} 0.0574+0.4967\mathrm{i} \\ 0.2846-0.0486\mathrm{i} \\ 0.2846-0.0486\mathrm{i} \\ -0.0640-0.2815\mathrm{i} \\ 0.2846-0.0486\mathrm{i} \\ -0.0640-0.2815\mathrm{i} \\ -0.0640-0.2815\mathrm{i} \\ -0.4809+0.1370\mathrm{i} \end{pmatrix}.
$$

These POVMs, combined with the following estimator coefficients

$$
\begin{gathered}
\xi_{x1} = -0.1338,\ \ \xi_{x2} = 0.1338,\ \ \xi_{x3} = -2.4681,\ \ \xi_{x4} = 0.7817, \\
\xi_{x5} = -0.7817,\ \ \xi_{x6} = 0.7156,\ \ \xi_{x7} = -0.7156,\ \ \xi_{x8} = 2.4681
\end{gathered} \tag{26}
$$

and

$$
\begin{gathered}
\xi_{y1} = -2.4680,\ \ \xi_{y2} = 2.4680,\ \ \xi_{y3} = 0.1338,\ \ \xi_{y4} = 0.7157, \\
\xi_{y5} = -0.7157,\ \ \xi_{y6} = -0.7819,\ \ \xi_{y7} = 0.7819,\ \ \xi_{y8} = -0.1338\ ,
\end{gathered} \tag{27}
$$

give rise to a variance of 12.719. There is an extremely small difference of 0.003 rad^2 in the variance of this measurement and the three-copy Nagaoka bound, but this is not due to numerical error. Indeed, we were able to find a 10 outcome

POVM with a variance equal to the three-copy Nagaoka bound (up to numerical error $\approx 10^{-6}$). However, in the main text, the near-optimal 8 outcome POVM was implemented, because this does not require any ancilla qubits, simplifying the experimental realisation.

In the main text we also presented simulations based on a projective four-copy measurement, which theoretically surpasses the three-copy Nagaoka bound. The details of this POVM are found in our publicly available repository [8]. The four-copy Nagaoka bound on the variance is 12.368 rad^2. Our four-copy projective measurement attains a variance of 12.508 rad^2, which is below the three-copy Nagaoka bound, but does not saturate the four-copy Nagaoka bound. Due to computational restraints, we only searched for projective four-copy measurements, so saturating the four-copy Nagaoka bound may still be possible with a more general four-copy POVM.

Supplementary Note 4 . Experimental implementation of optimal POVMs

1. *Single- and two-copy POVM implementation*

In this Supplementary Note we describe how to convert the optimal POVMs into experimentally realisable quantum circuits. The quantum processors used in our experiments make measurements in the z basis. Therefore, in order to implement the POVMs it is necessary to diagonalise them in this basis. For example, for the two qubit POVM, we aim to find a unitary matrix U such that

$$U\begin{bmatrix}|\psi_1\rangle & |\psi_2\rangle & |\psi_3\rangle & |\psi_4\rangle\end{bmatrix} , \tag{28}$$

is diagonal in the computational basis, where $\begin{bmatrix}|\psi_1\rangle & |\psi_2\rangle & |\psi_3\rangle & |\psi_4\rangle\end{bmatrix}$ is a 4×4 matrix. Implementing this unitary matrix and measuring in the z basis is equivalent to implementing the desired POVM.

We start with the single qubit POVM for estimating θ_x, Eq. (16). We write

$$|\psi_{x1}\rangle = \frac{1}{\sqrt{2}}(-\mathrm{i}\,|0\rangle + |1\rangle) \qquad \text{and} \qquad |\psi_{x2}\rangle = \frac{1}{\sqrt{2}}(\mathrm{i}\,|0\rangle + |1\rangle) , \tag{29}$$

so that we wish to diagonalise $\begin{bmatrix}|\psi_{x1}\rangle & |\psi_{x2}\rangle\end{bmatrix}$. It is easy to verify that this is diagonalised by the following unitary matrix

$$U_x = \frac{1}{\sqrt{2}}\begin{pmatrix}\mathrm{i} & 1\\ -\mathrm{i} & 1\end{pmatrix} . \tag{30}$$

In Fig. 1 of the main text, single qubit unitary matrices are shown as green boxes in the circuit diagram. We use the following definition for the most general single qubit gate characterised by three parameters, θ, ϕ and λ

$$U_{1\mathrm{q}}(\theta,\phi,\lambda) = \begin{pmatrix}\cos(\frac{\theta}{2}) & -e^{\mathrm{i}\lambda}\sin(\frac{\theta}{2})\\ e^{\mathrm{i}\phi}\sin(\frac{\theta}{2}) & e^{\mathrm{i}(\lambda+\phi)}\cos(\frac{\theta}{2})\end{pmatrix} . \tag{31}$$

With this definition, up to a global phase factor, we can write $U_x = U_{1\mathrm{q}}(3\pi/2, 0, 3\pi/2)$. Following the same approach, to implement the POVM for estimating θ_y, Eq. (17), we require the following unitary matrix

$$U_y = \frac{1}{\sqrt{2}}\begin{pmatrix}1 & 1\\ -1 & 1\end{pmatrix} . \tag{32}$$

This can be implemented as $U_y = U_{1\mathrm{q}}(3\pi/2, 0, 0) = R_y(3\pi/2)$.

Optically these unitary matrices are implemented through a motorised quarter-wave plate (QWP) followed by a half-wave plate (HWP) and then another QWP. The HWP and QWP set to a specific angle θ implement the following

unitary transformations

$$\mathrm{HWP}(\theta) = e^{-\mathrm{i}\pi/2} \begin{pmatrix} \cos(\theta)^2 - \sin(\theta)^2 & 2\cos(\theta)\sin(\theta) \\ 2\cos(\theta)\sin(\theta) & -\cos(\theta)^2 - \sin(\theta)^2 \end{pmatrix} , \tag{33}$$

and

$$\mathrm{QWP}(\theta) = e^{-\mathrm{i}\pi/4} \begin{pmatrix} \cos(\theta)^2 + \mathrm{i}\sin(\theta)^2 & (1-\mathrm{i})\cos(\theta)\sin(\theta) \\ (1-\mathrm{i})\cos(\theta)\sin(\theta) & \mathrm{i}\cos(\theta)^2 + \sin(\theta)^2 \end{pmatrix} . \tag{34}$$

To implement U_x, we use the following angles QWP($-\pi/4$)HWP($-3\pi/4$)QWP($\pi/2$), which is equivalent to U_x up to a global phase shift. To implement U_y we use QWP(0.788913939)HWP(-1.174581359)QWP(0.003515755). This is not an exact implementation, but the numerical error in the implementation of U_y is significantly smaller than the expected MSE, hence can be ignored.

For the two-copy POVM, Eq. (20), note that $\langle\psi_1|$, $\langle\psi_2|$, $\langle\psi_3|$ and $\langle\psi_4|$ are orthonormal hence the unitary matrix which diagonalises $[|\psi_1\rangle, |\psi_2\rangle, |\psi_3\rangle, |\psi_4\rangle]$ is $[\langle\psi_1|, \langle\psi_2|, \langle\psi_3|, \langle\psi_4|]^T$. Any two qubit unitary matrix can be implemented by three CNOT gates, four arbitrary single qubit unitary matrices, Eq. (31), and three single qubit rotations [9]. This is the circuit shown in Fig. 1 (f) in the main text. The rotation parameters needed to implement this unitary matrix to an accuracy of 1 part in 10 million are given in Tables. S1 and S2.

	U_1	U_2	U_3	U_4
θ	1.332047081999419	1.332047066929274	2.328216659679420	0.813375851824116
ϕ	0.896361779524331	0.674434373615608	1.509056929793133	0.461859523404381
λ	0.228222830422120	1.342573324008889	2.257150772710729	-0.686354414697494

TABLE S1. Parameters required to implement the arbitrary single qubit unitary matrices for the optimal two-copy circuit in Fig. 1 (f) from the main text.

	R_y	R_y	R_z
θ	0.505232912711076	-0.705293075042498	1.378157349320011

TABLE S2. Parameters required to implement the single qubit rotations for the optimal two-copy circuit in Fig. 1 (f) from the main text.

2. *State preparation*

The final step before running these optimal measurement circuits is to prepare the correct probe state. For a given decoherence strength ϵ and rotation angles θ_x and θ_y we need to prepare three different states per qubit with the appropriate probabilities. The state $|\psi_\theta\rangle = R_y(\theta_y)R_x(\theta_x)|0\rangle$ is prepared with probability $1-\epsilon$ and the states $|0\rangle$ and $|1\rangle$ are prepared with probability $\epsilon/2$ for each qubit. For implementing collective measurements, each two qubit state is a Kronecker product of two states and is prepared with a probability equal to the product of the probabilities for each individual state. For example, the state $|\psi_\theta\rangle \otimes |0\rangle$ is prepared with probability $(1-\epsilon)\epsilon/2$.

In principle, there should be no error in the value of ϵ used in our experiments, as the exact probability with which we prepare each state is known. However, in practice, due to non-zero state preparation error, there will be some error in the value of ϵ used. We now estimate the error in ϵ, σ_ϵ, given a certain state preparation error. We ignore readout error when calculating σ_ϵ as this does not affect the actual state we prepare. For simplicity, we will consider the case when $\theta_x = \theta_y = 0$, so that the state we wish to prepare is $(1-\epsilon/2)|0\rangle\langle 0| + \epsilon/2|1\rangle\langle 1|$. Using N qubits for

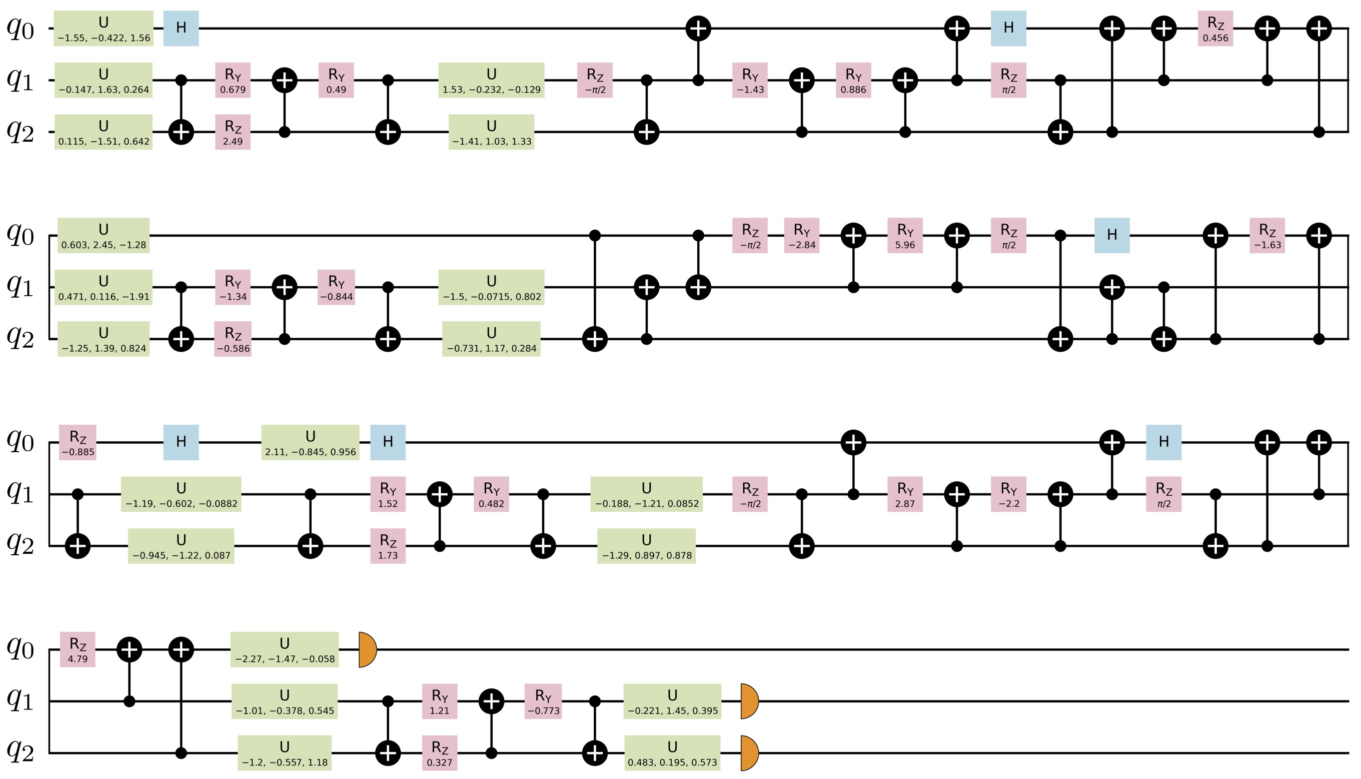

FIG. S1. **Three-copy measurement circuit which surpasses the two-copy Nagaoka bound.** Circuit reads from left to right and continues from top to bottom. In total the circuit requires 43 CNOT gates, 20 arbitrary single qubit unitary matrices, 6 Hadamard gates and 28 single qubit rotations, a total of 88 free parameters to optimise. The gates labelled U are as defined in Eq. (31). The circuit diagram was constructed using the IBM Q QISKIT package.

the complete experiment, the true ϵ value is given by

$$\epsilon = 2 - \frac{2N_{0,\text{prep}}}{N} \ , \tag{35}$$

where $N_{0,\text{prep}}$ is the number of $|0\rangle$ states which are actually prepared. We assume that the state preparation error is symmetric for preparation of both the $|0\rangle$ and $|1\rangle$ state, which we denote p_p. This is the probability of initialising the wrong qubit state. We can then write ϵ in terms of the number of $|0\rangle$ and $|1\rangle$ states which would ideally be prepared and the probability of incorrect initialisation

$$\epsilon = 2 - \frac{2N(1-\epsilon_\text{i}/2)(1-p_p)}{N} - \frac{2Np_p\epsilon_\text{i}/2}{N} \ , \tag{36}$$

where ϵ_i is the ϵ value which would be prepared in an ideal experiment. Finally, assuming that the $|0\rangle$ and $|1\rangle$ initialisation errors are independent, we find the variance of ϵ as

$$\sigma_\epsilon^2 = \frac{4}{N} p_p(1-p_p) \ . \tag{37}$$

As we average our results over 400 repetitions of the same experiment, σ_ϵ^2 is decreased by a further factor of 400. Using the calibration data for our devices, we find that σ_ϵ is on the order 10^{-4} or smaller for all devices, hence horizontal error bars on Fig 2 (e) in the main text would be negligible and we do not include them.

3. *Three- and four-copy POVM implementation and noisy simulation*

In order to implement the three-copy POVM presented in the Supplementary Note 3 we need a unitary matrix which diagonalises $\left[\left|\psi_1^{3c}\right\rangle, \left|\psi_2^{3c}\right\rangle, \left|\psi_3^{3c}\right\rangle, \left|\psi_4^{3c}\right\rangle, \left|\psi_5^{3c}\right\rangle, \left|\psi_6^{3c}\right\rangle, \left|\psi_7^{3c}\right\rangle, \left|\psi_8^{3c}\right\rangle\right]$. This POVM is constructed such that the $\left|\psi_i^{3c}\right\rangle$ are orthonormal. Therefore, the unitary matrix to be implemented experimentally is

$$\left[\left|\psi_1^{3c}\right\rangle, \left|\psi_2^{3c}\right\rangle, \left|\psi_3^{3c}\right\rangle, \left|\psi_4^{3c}\right\rangle, \left|\psi_5^{3c}\right\rangle, \left|\psi_6^{3c}\right\rangle, \left|\psi_7^{3c}\right\rangle, \left|\psi_8^{3c}\right\rangle\right]^\dagger .$$

Using the technique presented in Ref. [10], we are able to convert this unitary matrix to a three qubit quantum circuit, shown in Fig. S1. This circuit consists of 43 CNOT gates, 20 single qubit unitary matrices, 6 Hadamard gates and 28 single qubit rotations, and is an extensive circuit. When implemented on various superconducting processors, the results of this measurement did not reach the theoretical limits. This can possibly be attributed to the gate error rates of the different processors or crosstalk between qubits. The quantum circuit corresponding to our four-copy POVM can be found in our publicly available repository [8]. The decomposition of the four-copy measurement which we use contains 115 CNOT gates, hence unsurprisingly the four-copy POVM cannot reach the theoretical limits with even small amounts of noise. In Fig. 2 (f) in the main text we simulated implementing these measurements on noisy quantum computers. This simulation used the IBM Q QISKIT package noise models and noise was modelled as depolarising noise for different gate error rates. The results of this simulation suggest that with realistic future noise levels, quantum processors may be able to implement three-copy measurements with a precision approaching the theoretical limits. Four-copy measurements, on the other hand, will require considerably lower gate error rates to reach the theoretical limits. Although this discussion comes with the caveat that modelling noise in such a complex system is unlikely to be overly accurate, we do observe good qualitative agreement between simulation and experiment for the three-copy measurement.

The results presented for the noisy simulations in Fig. 2 (f) in the main text use a slightly different error mitigation method than that used for the experimental data. This is done to avoid erroneously predicting small variances from our noisy simulations. In Fig. S2 we plot the predicted values of θ_x using our four-copy measurement as a function of the input θ, simulated for two different gate error rates. Qualitatively, it is clear that as the noise is increasing the estimator is predicting values closer to 0. Hence, we would expect such an estimator to perform worse with increasing gate error rate. Quantitatively however, without any error mitigation, the MSE actually decreases with increasing gate error rate for $\theta = 0$. With gate error rates of 1×10^{-3}, the MSE is 11.89 rad^2, for gate error rates of 5×10^{-3}, the MSE is 11.71 rad^2. The fact that higher gate error rates give a lower MSE indicates something is not correct, but even more alarming is that both of these MSEs are below what is allowed by the Holevo bound. The reason for this is that the high gate error rate biases the estimator. For an input angle of $\theta = 0$, an estimator which predicts $\hat{\theta} = 0$ every time will have a MSE of 0 rad^2. Hence, for our noisy simulations, the calibration model we use corrects for both the gradient of the estimator and the offset, $\hat{\theta}_x = m_x \hat{\theta}_{\text{noisy},x} + c_x$. As we will stress in Supplementary Note 5 , a calibration model of this form can actually bias the estimator itself. Hence, we only use this form of calibration for our noisy simulation results. This is more a reflection of the limitations of our simulations than anything else. It is likely that a more comprehensive noise model is needed to completely capture the noise of a real quantum processor.

The results of our three- and four-copy collective measurements show the trade-off between what is gained by implementing a theoretically better measurement versus what is lost by the increased experimental complexity of such a measurement. However, this work may be viewed as one small step towards implementing collective measurements on a large number of copies of the probe state simultaneously. For comparison, we will now briefly discuss alternative approaches to implementing collective measurements. All previous approaches have been restricted to implementing collective measurements on two copies of the probe state [11–15] and have relied on optical systems. Owing to the way that two copies of the quantum state were created in these approaches, it is difficult to extend to measurements on more than two copies of the quantum state simultaneously. Experiments using three degrees of freedom of a single

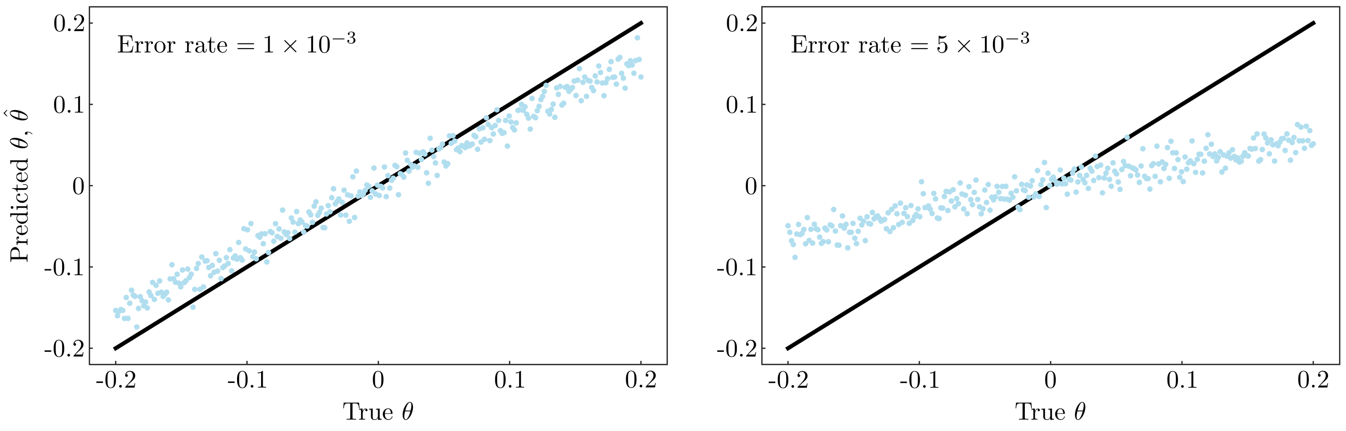

FIG. S2. **Noisy simulation of our four-copy measurement under depolarising noise.** Shown are the predicted values of θ_x using our four-copy measurement as a function of the true θ, simulated for two different gate error rates. The black line shows the true θ value.

photon may offer a way around this problem [16]. Another possible way of implementing collective measurements on more than two copies of a quantum state simultaneously is through quantum walks. Quantum walks were originally proposed and demonstrated for implementing POVMs on single qubit states [17–19] and it was a very similar technique which has enabled some of the recent demonstrations of two-copy collective measurements [13–15]. The theory of quantum walks as a measurement tool has been extended to POVMs on qudit states [20]. This, combined with recent advances in optical quantum state engineering [21, 22], may some day allow optical approaches to collectively measure more than two copies of the quantum state simultaneously. It is likely that the continued development of collective measurements on multiple platforms will be useful.

Supplementary Note 5 . Effect of error mitigation on the bias of an estimator

When calculating the various bounds in Supplementary Note 1 it was assumed that the rotations to be estimated are small, meaning the estimators are unbiased exactly at $\theta = 0$. It is not guaranteed that the estimators will remain unbiased away from $\theta = 0$. Fig. S3 shows the predicted angles, $\hat{\theta}$, for a range of different input angles, θ. Evidently, the estimator remains approximately unbiased for a large range of θ. It is important for any estimator to be unbiased to ensure a fair comparison is being made. Provided we have sufficiently many probe states available, it is always possible to operate in the region where the estimator is unbiased. This can be done by taking a small sample of the probe states, $\sqrt{N} \ll N$, where N is the number of available probe states, to obtain a rough estimate of θ. The measurement apparatus can then be adjusted to operate in the unbiased region by taking into account this rough estimate.

In this work error mitigation was used to allow us to observe quantum-enhanced metrology. An important requirement on any error mitigation technique used is that it does not introduce any bias into the estimator. We primarily focused on Clifford data regression error mitigation [23] which essentially amounts to producing a model for the system being interrogated. In the main text we used a model of the form

$$\hat{\theta}_{x(y)} = \hat{\theta}_{\text{noisy, } x(y)} + c_{x(y)} \ , \tag{38}$$

where $c_{x(y)}$ is a constant which accounts for the offset of the true estimate from the noisy estimate. However, the authors who introduced this form of error mitigation originally proposed a linear model [23] of the form

$$\hat{\theta}_{x(y)} = m_{x(y)} \hat{\theta}_{\text{noisy, } x(y)} + c_{x(y)} \ , \tag{39}$$

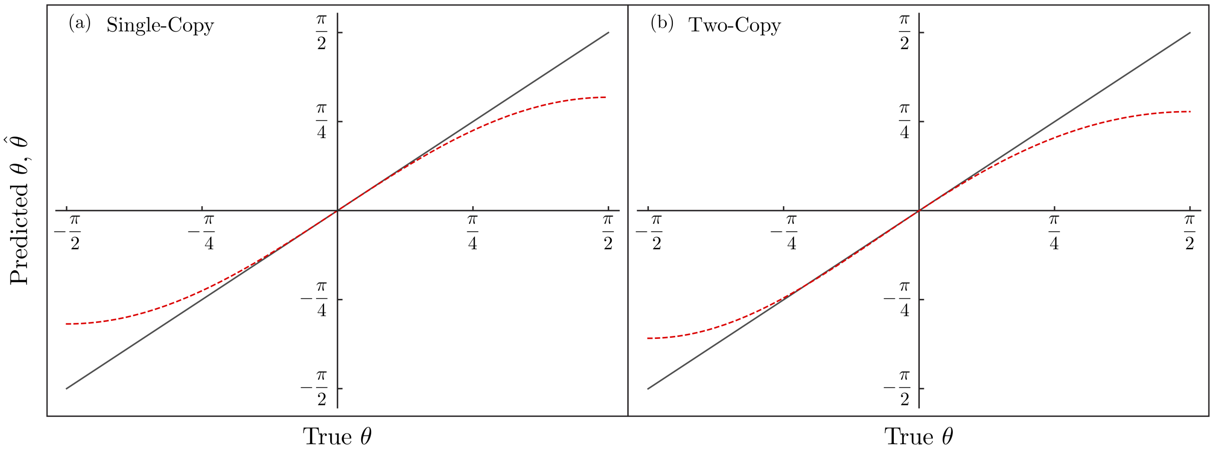

FIG. S3. **Predicted θ_x values.** For θ close to 0 the estimator is unbiased, i.e. $\hat{\theta} = \theta$. However, for larger θ the estimator is no longer unbiased. This is to be expected as the estimator was constructed to be unbiased in the region around $\theta = 0$. (a) and (b) show results for the single-copy and two-copy estimator respectively. Results for estimating θ_x and θ_y are similar. The black line shows the true θ values.

where the extra term $m_{x(y)}$ is another constant, determining the slope of the model. Given that the linear model proposed is motivated by depolarising noise, it seems a natural choice. While this may be true for many other applications of quantum processors, it is not true for quantum metrology. For quantum metrology $\hat{\theta}_{\text{noisy, }x(y)}$ will be some distribution of estimated angles with a certain variance. If we were to multiply this distribution by some constant $m_{x(y)}$ which is less than 1, we would artificially reduce the variance of the estimator. It then becomes possible to have variances which appear smaller than the minimum allowed by quantum mechanics. The effect of naively applying error mitigation based on Eq. (39) to quantum metrology is shown in Fig. S4. In this example the fitted gradient depends on the value of ϵ: for larger ϵ, m is smaller. As Fig. S4 shows, with this error mitigation model, we apparently surpass the two-copy Nagaoka bound in some regions, whereas, with an unbiased estimator this is not possible. Thus, it is clear that error mitigation cannot be naively applied to quantum metrology. Other error mitigation techniques were investigated, including zero noise extrapolation [24–26] and quantum readout-error mitigation [27, 28], however they were not found to be as effective as Clifford data regression. Specifically, zero noise extrapolation introduces a large overhead, which is not ideal for quantum metrology, and quantum readout-error mitigation offered only marginal improvements in the MSE. Further study is needed to fully understand what error mitigation protocols are best for quantum metrology. Indeed, recent error mitigation proposals, specifically designed for quantum metrology, may prove more effective [29].

Supplementary Note 6 . Further three-copy measurement results

Fig. 2 in the main text shows the results of our three-copy measurement implemented on the Rigetti Aspen-9 and F-IBM QS1 processors. Although these measurements did not reach the theoretical limit, the MSE for the F-IBM QS1 processor is within a factor of 2 of this limit. This perhaps suggests that with minor improvements in gate error rates, the theoretical limits on three-copy measurements may be approached. However, as we now show, this is not necessarily true. We estimated a range of angles using the three-copy measurement on several different quantum processors. The results of this are shown in Fig. S5, where it is evident that the three-copy measurements are effectively useless at distinguishing different angles. For all devices tested, there was no meaningful correlation between the input angle and the estimated angle. This is in stark contrast to the single-copy and two-copy measurements, shown in

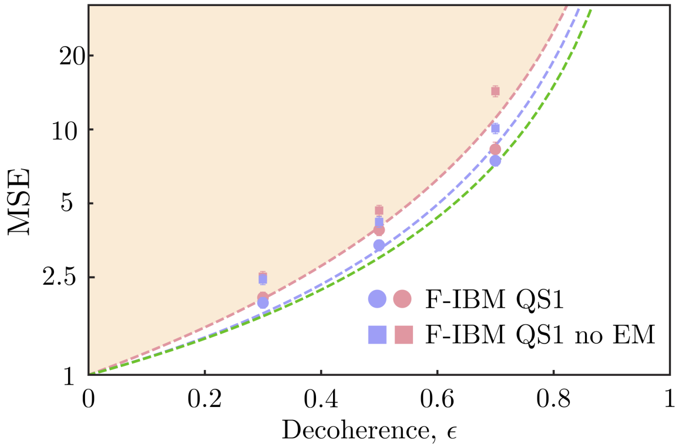

FIG. S4. **Effect of naive error mitigation on estimation performance.** We show the same results as presented in Fig. 2 (e) of the main text for the IBM Q System One device, however now the error mitigation (EM) is performed using the model given by Eq. (39), instead of the model used in the main text, Eq. (38). Data is presented for $\epsilon = 0.3$, 0.5 and 0.7. We show the MSE obtained both with and without error mitigation. For $\epsilon = 0.3$ the MSE is now considerably larger than in the main text, while for $\epsilon = 0.7$ it is considerably reduced. This can be explained by the fitted value of $m_{x(y)}$. As in the main text the dashed pink, purple and green lines correspond to the single-copy Nagaoka, two-copy Nagaoka and Holevo bounds respectively.

Figs. 1 and 2 in the main text. The data-set measured for the Rigetti Aspen-9 device differs slightly from the IBM Q devices, due to different device accessibility. Further theoretical and experimental studies will be required to fully understand the utility of three-copy collective measurements for metrology.

Supplementary Note 7 . Problem where collective measurements on many copies of the probe state are necessary

For the problem considered in this work, two-copy measurements are able to achieve almost the same precision as the Holevo bound. Performing collective measurements on three or more copies of the probe state offers only marginal improvements in the precision at the cost of greater experimental complexity. Thus, it is natural to wonder if it is truly necessary to implement collective measurements on more than two copies of the quantum state. In this Supplementary Note we provide an example which clearly shows that collective measurements on many copies of the quantum state are necessary.

We examine a similar problem to the main text, estimating qubit rotations subject to the amplitude damping channel, using the probe state $|1\rangle$. After the rotations and amplitude damping, the probe and its derivatives are given by

$$\rho = \begin{pmatrix} \mathrm{p} & 0 \\ 0 & 1-\mathrm{p} \end{pmatrix}, \qquad \partial_x \rho = \frac{\mathrm{i}\sqrt{1-\mathrm{p}}}{2} \begin{pmatrix} 0 & -1 \\ 1 & 0 \end{pmatrix}, \qquad \text{and} \qquad \partial_y \rho = \frac{\sqrt{1-\mathrm{p}}}{2} \begin{pmatrix} 0 & -1 \\ -1 & 0 \end{pmatrix}, \tag{40}$$

where p is the amplitude damping strength. The following matrices then optimise the Holevo and Nagaoka bounds

$$X_x = \frac{\mathrm{i}}{\sqrt{1-\mathrm{p}}} \begin{pmatrix} 0 & -1 \\ 1 & 0 \end{pmatrix} \qquad \text{and} \qquad X_y = \frac{1}{\sqrt{1-\mathrm{p}}} \begin{pmatrix} 0 & -1 \\ -1 & 0 \end{pmatrix}. \tag{41}$$

By direct substitution it can be verified that these matrices satisfy the unbiased conditions, Eq. (5). The corresponding

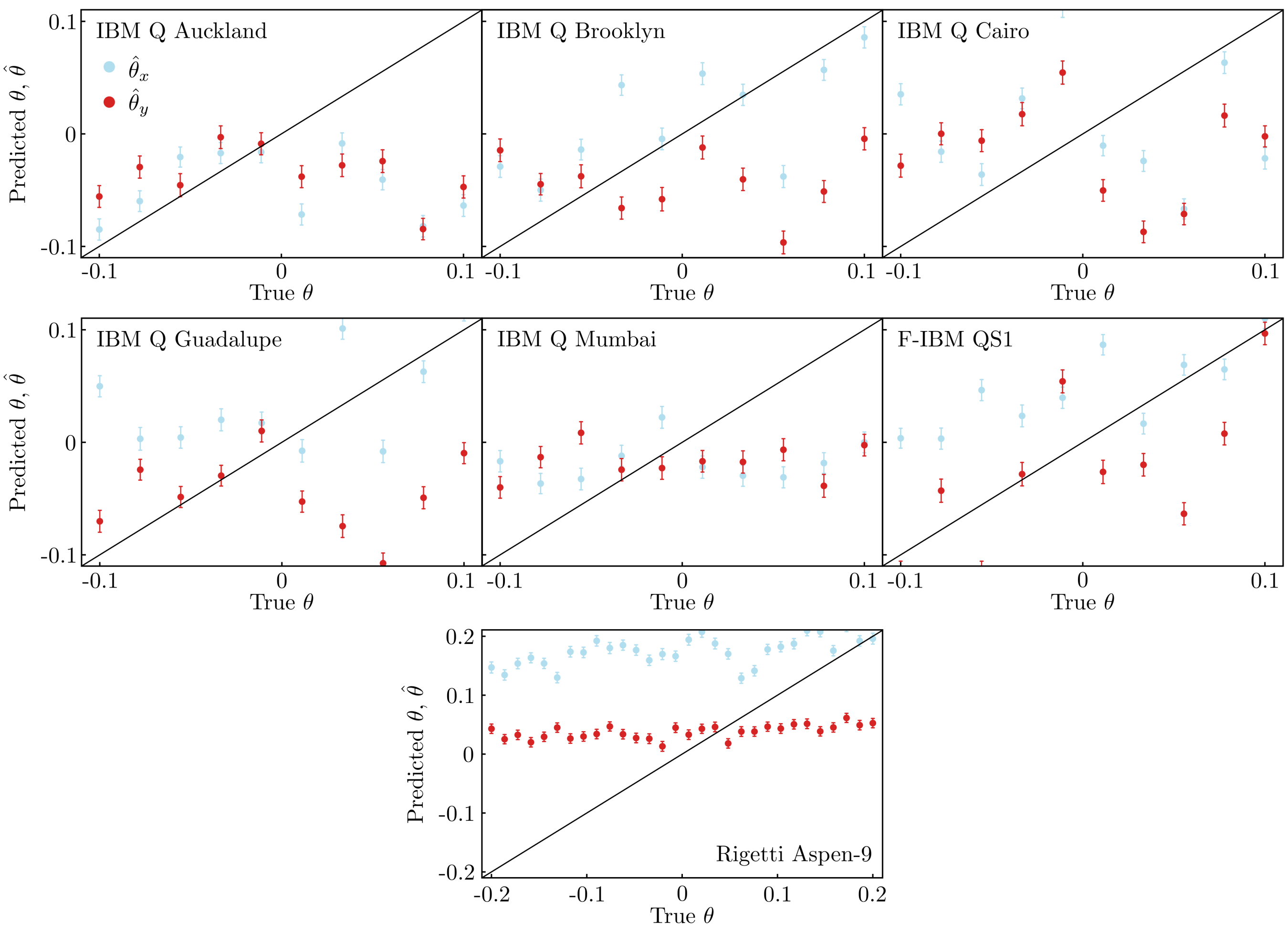

FIG. S5. **Three-copy measurement implemented on several quantum processors.** We show the estimated θ_x and θ_y values, predicted by the three-copy measurement, as a function of the true angle θ, shown as the black line. For all devices, there is no meaningful correlation between the true and predicted values, rendering them effectively useless as estimators. All data points are based on at least 17,000 shots and all error bars correspond to one standard deviation obtained through bootstrapping.

Holevo bound is given by

$$\mathcal{H}_{\mathrm{AD}} = \begin{cases} 4 & \text{for } \ \mathrm{p} \leq 1/2\ , \\ \frac{4\mathrm{p}}{1-\mathrm{p}} & \text{for } \ \mathrm{p} > 1/2\ . \end{cases} \quad . \tag{42}$$

The single-copy Nagaoka bound is given by

$$\mathcal{N}_{1,\mathrm{AD}} = \frac{4}{1-\mathrm{p}}\ . \tag{43}$$

Considering measurements on two copies of the probe state simultaneously, the following matrices optimise the two-copy Nagaoka bound

$$X_x = \frac{\mathrm{i}}{2\sqrt{1-\mathrm{p}}} \begin{pmatrix} 0 & -1 & -1 & 0 \\ 1 & 0 & 0 & -1 \\ 1 & 0 & 0 & -1 \\ 0 & 1 & 1 & 0 \end{pmatrix} \qquad \text{and} \qquad X_y = \frac{-1}{2\sqrt{1-\mathrm{p}}} \begin{pmatrix} 0 & 1 & 1 & 0 \\ 1 & 0 & 0 & 1 \\ 1 & 0 & 0 & 1 \\ 0 & 1 & 1 & 0 \end{pmatrix}\ . \tag{44}$$

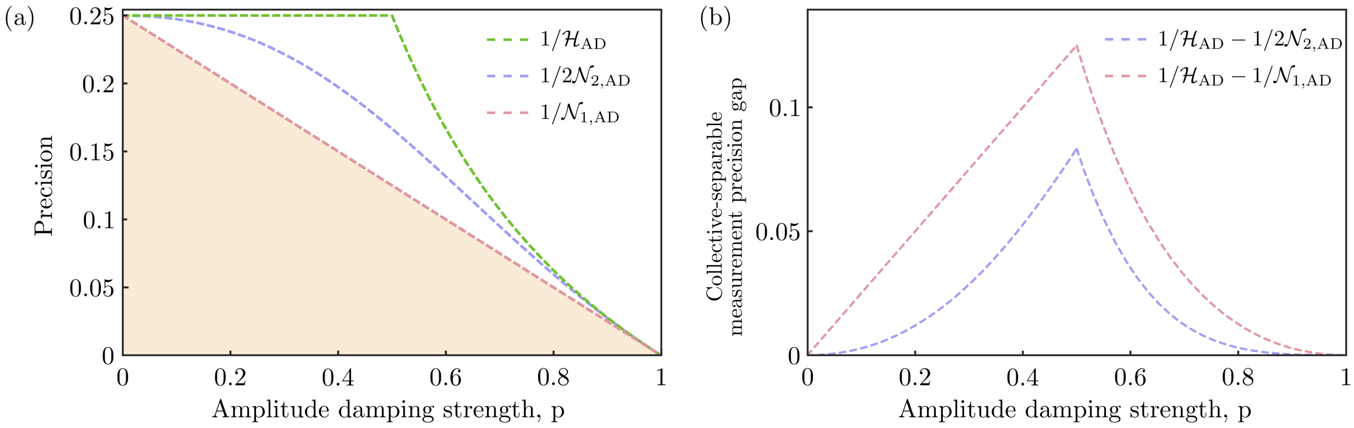

FIG. S6. **Attainable precision with separable and collective measurements in the amplitude damping channel.** (a) Inverse of the single-copy Nagaoka, two-copy Nagaoka and Holevo bounds as a function of the amplitude damping strength p. Although the two-copy Nagaoka bound is closer to the Holevo bound that the single-copy Nagaoka bound, there still remains a large gap between the two-copy Nagaoka and Holevo bounds. (b) The difference between the Holevo and Nagaoka bounds as a function of the amplitude damping strength. The large gap between the two-copy Nagaoka bound and the Holevo bound can only be narrowed by collective measurements on more than two copies of the probe state.

The two-copy Nagaoka bound is given by

$$2\mathcal{N}_{2,\rm AD} = \frac{4}{1-\rm p} - 4\rm p \ , \tag{45}$$

where we include the factor of two to account for the resources used.

The inverse of the single-copy Nagaoka, two-copy Nagaoka and the Holevo bounds are shown in Fig. S6 (a). Although the two-copy Nagaoka bound is closer to the Holevo bound, there remains a considerable gap between these two bounds. The difference between both Nagaoka bounds and the Holevo bound is shown in Fig. S6 (b). For this example, it is evident that measurements on more than two copies of the probe state will be required if the ultimate limits in quantum metrology are to be attained. This is also a physically relevant channel, as the amplitude damping channel can be used to model the decay of an atom from its excited state to its ground state. We can expect that many other tasks in quantum information will require collective measurements on many copies of the probe state.

Supplementary Note 8 . Computing Lu and Wang's metrological bound based on Heisenberg's uncertainty principle

Recently Lu and Wang derived a trade-off relation between measurement variances when estimating two parameters based on uncertainty relations [30]. As in the main text, we shall refer to this bound as the LW uncertainty relation. We now compute the LW uncertainty relation for the problem considered in the main text. To do so we first compute the symmetric logarithmic derivative (SLD) quantum Fisher information matrix $\mathcal{F}$,

$$\mathcal{F}_{jk} = \mathrm{Re}(Q_{jk}) = \mathrm{Re}(\mathrm{Tr}[L_j L_k \rho]) \ , \tag{46}$$

where L_j satisfies $(L_j\rho + \rho L_j)/2 = \partial_j \rho$. It is easily verified that the matrix Q is given by

$$Q = \begin{pmatrix} (1-\epsilon)^2 & \mathrm{i}(1-\epsilon)^3 \\ -\mathrm{i}(1-\epsilon)^3 & (1-\epsilon)^2 \end{pmatrix} \ . \tag{47}$$

Based on this the $\tilde{c}_{jk}$ terms (Eq. (7) of Ref. [30]) can be computed as $\tilde{c}_{11} = \tilde{c}_{22} = 0$ and $\tilde{c}_{12} = \tilde{c}_{21} = 1$. These terms then allow the following metrological bound to be derived (Eq. (8) of Ref. [30])

$$\frac{1}{v_x} + \frac{1}{v_y} \leq (1-\epsilon)^2 \, . \tag{48}$$

The LW uncertainty relation provides a tradeoff relation between the variances which can be attained for estimating the two rotation angles. The minimum total variance is achieved when $v_x = v_y = 2/(1-\epsilon)^2$, which coincides with the single-copy Nagaoka bound. Therefore, our two-copy collective measurement, which surpasses the single-copy Nagaoka bound, can also surpass the LW uncertainty relation. To the best of our knowledge, this is the first time that a "universally valid" uncertainty principle has been surpassed, albeit indirectly, i.e. through measurement variances as opposed to directly probing the observables.

The LW uncertainty relation is based on previously derived measurement uncertainty relations which explicitly assume that only separable measurements are used. These previous bounds are based on the usual notion of uncertainty relations for operators, whereas Lu and Wang were the first to map this to quantum parameter estimation. It is possible to calculate a two-copy version of the LW uncertainty relation, however this is unsatisfying as it gives a bound which cannot be reached even with collective measurements on infinitely many copies of the probe state. In Supplementary Note 11 we present one possible way to modify the LW uncertainty relation so that it accounts for collective measurements.

Supplementary Note 9 . POVMs violating the LW uncertainty relation with unbalanced variances

Fig. 3 in the main text shows single-copy measurements which verify the LW relation when restricted to separable measurements for a range of v_x and v_y values. For $v_x = v_y$, this is achieved using the single-copy POVMs presented in Supplementary Note 2 , with an equal number of qubits used for estimating θ_x and θ_y. For $v_x \neq v_y$ the same POVM is used, but now a different number of qubits are used for estimating θ_x and θ_y. The total number of qubits used in each experiment remains fixed. By assigning more qubits to estimating θ_x, we can reduce v_x at the expense of increasing v_y and vice versa.

For the measurements violating the LW uncertainty relation, the two-copy measurement in Supplementary Note 2 is sufficient for the point where $v_x = v_y$. However, for the points where $v_x \neq v_y$, a new POVM is required. The purple line in Fig. 3 is obtained from the weighted Nagaoka bound, i.e. the two-copy Nagaoka bound computed with non-identity weight matrices. Changing the weight matrix corresponds to a transformation of the parameters being estimated [31]. Hence, after computing the weighted Nagaoka bound, we transform from the weighted variances $w_x v_x$ and $w_y v_y$ to the variances in the parameters of interest v_x and v_y. Each different weight matrix produces a line in the $v_x - v_y$ plane. The purple curve is then the envelope of all these lines. The line corresponding to the weighted Holevo bound is calculated similarly.

Similarly, when finding measurements where $v_x \neq v_y$, which violate the LW uncertainty relation, we find a measurement which minimises $w_x v_x + w_y v_y$. For $w_x = 1.4$, $w_y = 0.6$, we use the POVM $\{\Pi_i^{w1} = |\psi_i^{w1}\rangle\langle\psi_i^{w1}|\}$, where

$$\begin{aligned} |\psi_1^{w1}\rangle &= \begin{pmatrix} 0.2813 - 0.5023\mathrm{i} \\ 0.4284 + 0.0308\mathrm{i} \\ 0.4284 + 0.0308\mathrm{i} \\ 0.2318 + 0.4958\mathrm{i} \end{pmatrix}, \quad |\psi_2^{w1}\rangle = \begin{pmatrix} 0 \\ -0.6664 - 0.2366\mathrm{i} \\ 0.6664 + 0.2366\mathrm{i} \\ 0 \end{pmatrix}, \\ |\psi_3^{w1}\rangle &= \begin{pmatrix} -0.5584 - 0.1590\mathrm{i} \\ 0.3481 + 0.0991\mathrm{i} \\ 0.3481 + 0.0991\mathrm{i} \\ -0.6089 - 0.1734\mathrm{i} \end{pmatrix}, \quad |\psi_4^{w1}\rangle = \begin{pmatrix} -0.3415 + 0.4635\mathrm{i} \\ 0.2048 + 0.3776\mathrm{i} \\ 0.2048 + 0.3776\mathrm{i} \\ 0.5473 + 0.0066\mathrm{i} \end{pmatrix}. \end{aligned} \tag{49}$$

This POVM, combined with the following estimator coefficients

$$\begin{aligned} &\xi^{w1}_{x,1} = -2.2810, \quad \xi^{w1}_{x,2} = 0, \quad \xi^{w1}_{x,3} = 0 \quad \text{and} \quad \xi^{w1}_{x,4} = 2.2810 \\ &\xi^{w1}_{y,1} = 1.5109, \quad \xi^{w1}_{y,2} = 0, \quad \xi^{w1}_{y,3} = -3.1423 \quad \text{and} \quad \xi^{w1}_{y,4} = 1.5109 \ , \end{aligned} \tag{50}$$

gives rise to the data point in Fig. 3 with reduced variance in estimating θ_x.

Similarly, for $w_x = 0.6$, $w_y = 1.4$, the necessary POVM is $\{\Pi^{w2}_i = |\psi^{w2}_i\rangle\langle\psi^{w2}_i|\}$, where

$$\begin{aligned} |\psi^{w2}_1\rangle &= \begin{pmatrix} 0.5530 + 0.1602\text{i} \\ 0.4243 - 0.0671\text{i} \\ 0.4243 - 0.0671\text{i} \\ 0.4304 - 0.3381\text{i} \end{pmatrix} , \quad |\psi^{w2}_2\rangle = \begin{pmatrix} 0.5515 + 0.1651\text{i} \\ -0.3202 - 0.2863\text{i} \\ -0.3202 - 0.2863\text{i} \\ 0.1784 + 0.5174\text{i} \end{pmatrix} , \\ |\psi^{w2}_3\rangle &= \begin{pmatrix} 0.2998 - 0.4973\text{i} \\ 0.3100 + 0.1869\text{i} \\ 0.3100 + 0.1869\text{i} \\ -0.3269 + 0.5422\text{i} \end{pmatrix} , \quad |\psi^{w2}_4\rangle = \begin{pmatrix} 0 \\ -0.6643 - 0.2423\text{i} \\ 0.6643 + 0.2423\text{i} \\ 0 \end{pmatrix} . \end{aligned} \tag{51}$$

The estimator coefficients for this POVM become

$$\begin{aligned} &\xi^{w2}_{x,1} = 1.5109, \quad \xi^{w2}_{x,2} = 1.5109, \quad \xi^{w2}_{x,3} = -3.1423 \quad \text{and} \quad \xi^{w2}_{x,4} = 0 \\ &\xi^{w2}_{y,1} = 2.2810, \quad \xi^{w2}_{y,2} = -2.2810, \quad \xi^{w2}_{y,3} = 0 \quad \text{and} \quad \xi^{w2}_{y,4} = 0 \ . \end{aligned} \tag{52}$$

This measurement gives rise to the data point in Fig. 3 with reduced variance in estimating θ_y.

Supplementary Note 10 . Measurement surpassing universally valid uncertainty relations for operators

Surpassing Lu and Wang's bound, should be equivalent to surpassing the measurement uncertainty relations for operators on which it is based. We now show this explicitly. The LW uncertainty relation is based on an uncertainty relation which was a cumulation of work from Ozawa and Branciard [32–35]. These works provided trade-off relations for measuring two Hermitian operators A and B. When A and B do not commute, they cannot be jointly measured and so instead we measure a pair of commuting observables $\mathcal{A}$ and $\mathcal{B}$ which approximate the ideal measurement. The approximate observables are measured on an extended Hilbert space of the quantum state ρ combined with an ancilla state η. The measurement errors for the ideal observables A and B are then given by

$$\epsilon_A = \sqrt{\text{Tr}[(\mathcal{A} - A \otimes \mathbb{I})^2(\rho \otimes \eta)]} \quad \text{and} \quad \epsilon_B = \sqrt{\text{Tr}[(\mathcal{B} - B \otimes \mathbb{I})^2(\rho \otimes \eta)]} \ . \tag{53}$$

The following uncertainty relation is then claimed to hold

$$\epsilon_A^2 \sigma_B^2 + \epsilon_B^2 \sigma_A^2 + 2\sqrt{\sigma_A^2 \sigma_B^2 - D_{AB}^2}\,\epsilon_A \epsilon_b \geq D_{AB}^2 \ , \tag{54}$$

where $\sigma_A = \sqrt{\text{Tr}[A^2\rho] - (\text{Tr}[A\rho])^2}$ and $D_{AB} = \text{Tr}[|\sqrt{\rho}(AB - BA)\sqrt{\rho}|]/2$ where $|X| = \sqrt{X^\dagger X}$. In order to map our measurements to this operator approach, we need the ideal operators A and B. For a parameter estimation problem these are always given by the SLD operators, $A = L_x$ and $B = L_y$. For the problem considered in this work, the SLD operators are

$$L_x = -(1-\epsilon)\sigma_y \quad \text{and} \quad L_y = (1-\epsilon)\sigma_x \ . \tag{55}$$

Substituting this in gives $\sigma_A = \sigma_B = 1 - \epsilon$. We can also evaluate D_{AB} as $(1-\epsilon)^2$. The uncertainty relation for this problem therefore becomes

$$\epsilon_A^2 + \epsilon_B^2 \geq (1-\epsilon)^2 \ . \tag{56}$$

At this point we can proceed in one of two ways to show that this relation is violated. From a metrological perspective, Lu and Wang define the "regret of the Fisher information" (hereafter abbreviated to regret), as the difference between the classical Fisher information (CFI) of a specified measurement F and the SLD quantum Fisher information $\mathcal{F}$. Then an equivalence is drawn between the regret and the measurement errors for the ideal observables $R_{xx} = \epsilon_A^2$ and $R_{yy} = \epsilon_B^2$. Therefore, by computing the CFI for of our measurements we can evaluate the regret and in turn the uncertainty relation, Eq. (56). Given a POVM $\{\Pi_m\}$ and a density matrix which depends on the parameters of interest $\rho(\theta_x, \theta_y)$, the CFI can be computed as

$$F_{j,k} = \sum_m \mathrm{Tr}[\rho\Pi_m] \frac{\partial \log(\mathrm{Tr}[\Pi_m\rho])}{\partial\theta_j} \frac{\partial \log(\mathrm{Tr}[\Pi_m\rho])}{\partial\theta_k} , \tag{57}$$

where log refers to the natural logarithm. Using the single- and two-copy measurements specified previously, Eqs. (13) and (20) respectively, we can evaluate the CFI for our two measurements as

$$\mathrm{CFI}_\rho = \frac{(1-\epsilon)^2}{2} \begin{pmatrix} 1 & 0 \\ 0 & 1 \end{pmatrix} , \tag{58}$$

and

$$\mathrm{CFI}_{\rho\otimes\rho} = \frac{2(1-\epsilon)^2}{4 - 2\epsilon + \epsilon^2} \begin{pmatrix} 1 & 0 \\ 0 & 1 \end{pmatrix} . \tag{59}$$

Note that the two-copy CFI has been scaled by a factor of two to account for the fact that twice as many resources are being used. Evaluating the regret and substituting into Eq. (56), shows that the uncertainty relation is saturated for the single-copy measurement and violated for the two-copy measurement.

Equivalently, we can give the exact forms for the approximate observables $\mathcal{A}$ and $\mathcal{B}$. For this we require the measurement channel $\Phi : S(\mathcal{H}_S) \to S(\mathcal{H}_R)$ introduced by Lu and Wang, which maps from the set of all density matrices on a Hilbert space $\mathcal{H}_S$ to density matrices on an alternative Hilbert space $\mathcal{H}_R$ which acts as a register of all the measurement outcomes. This channel is defined as

$$\Phi : \rho \mapsto \sum_m \mathrm{Tr}[\rho\Pi_m] \left|m\right\rangle \left\langle m\right| , \tag{60}$$

where $|m\rangle$ are states forming an orthonormal basis in $\mathcal{H}_R$. The new SLD operator for the density matrix $\Phi(\rho)$ is given by

$$\tilde{L}_j = \sum_m \frac{\partial \mathrm{Tr}[\rho\Pi_m]}{\partial\theta_j} \left|m\right\rangle \left\langle m\right| , \tag{61}$$

The approximate observables can then be defined as $\mathcal{A} = U^\dagger(\mathbb{I}_S \otimes \tilde{L}_x \otimes \mathbb{I}_R)U$ and $\mathcal{B} = U^\dagger(\mathbb{I}_S \otimes \tilde{L}_y \otimes \mathbb{I}_R)U$, where $\mathbb{I}_S$ and $\mathbb{I}_R$ are the identity matrices on the system and register Hilbert spaces respectively. U is a unitary matrix which satisfies

$$\Phi(\rho) = \mathrm{Tr}_{1,3}[U(\rho \otimes \eta \otimes \eta)U^\dagger] , \tag{62}$$

for all density matrices ρ, where η is any state in the register Hilbert space and $\mathrm{Tr}_{1,3}$ denotes the partial trace over the first and third systems.

We will now present unitary matrices which satisfy Eq. (62) for both the single-copy and two-copy measurements, allowing the approximate observables $\mathcal{A}$ and $\mathcal{B}$ to be obtained. For the single-copy measurement we first use the Naimark extension [7] to convert the measurement in Eq. (13) to projectors. One possible Naimark extension is

$$\left|\psi\right\rangle_{1,E} = \frac{1}{\sqrt{2}} \begin{pmatrix} 1 \\ 0 \\ \mathrm{i} \\ 0 \end{pmatrix} , \quad \left|\psi\right\rangle_{2,E} = \frac{1}{\sqrt{2}} \begin{pmatrix} 1 \\ 0 \\ -\mathrm{i} \\ 0 \end{pmatrix} , \quad \left|\psi\right\rangle_{3,E} = \frac{1}{\sqrt{2}} \begin{pmatrix} 0 \\ 1 \\ 0 \\ 1 \end{pmatrix} \quad \text{and} \quad \left|\psi\right\rangle_{4,E} = \frac{1}{\sqrt{2}} \begin{pmatrix} 0 \\ 1 \\ 0 \\ -1 \end{pmatrix} . \tag{63}$$

Using this projection we define the following unitary matrix

$$U_\Pi = |0\rangle \langle\psi|_{1,E} + |1\rangle \langle\psi|_{2,E} + |2\rangle \langle\psi|_{3,E} + |3\rangle \langle\psi|_{4,E} \ . \tag{64}$$

We next define

$$\begin{aligned} U_1 &= \mathbb{I}_2 \otimes C_{1,2} \otimes \mathbb{I}_4 \ , \\ U_2 &= U_\Pi \otimes \mathbb{I}_8 \ , \\ U_3 &= C_{1,4} \otimes \mathbb{I}_2 \quad \text{and} \\ U_4 &= \mathbb{I}_2 \otimes C_{1,4} \ , \end{aligned} \tag{65}$$

where $\mathbb{I}_d$ is the $d-$dimensional identity matrix and

$$\begin{aligned} C_{1,2} &= |0\rangle\langle 0| \otimes \mathbb{I}_2 + |1\rangle\langle 1| \otimes \sigma_x \quad \text{and} \\ C_{1,4} &= |0\rangle\langle 0| \otimes \mathbb{I}_8 + |1\rangle\langle 1| \otimes \mathbb{I}_4 \otimes \sigma_x \ . \end{aligned} \tag{66}$$

The necessary single-copy unitary matrix is $U_{1\mathrm{q}} = U_{\mathrm{swap}}.U_4.U_3.U_2.U_1$, where U_{swap} swaps modes 2 and 3 followed by modes 1 and 2.

The two-copy measurement presented in Eq. (20) is already a projective measurement, hence we do not need to invoke Naimark's theorem. The necessary unitary matrix for the two-copy measurement is very similar to the single-copy unitary matrix, however in the definition of U_Π we need to replace $|\psi\rangle_{j,E}$ with $|\psi\rangle_j$ from Eq. (20). The unitary matrix U_{swap} now needs to swap modes 2 and 3, followed by modes 1 and 2, followed by modes 3 and 4 and finally modes 2 and 3. For the single-copy and two-copy measurements the total ancilla systems are $|x_+\rangle\langle x_+| \otimes |z_+\rangle\langle z_+| \otimes |z_+\rangle\langle z_+| \otimes |z_+\rangle\langle z_+|$ and $|x_+\rangle\langle x_+| \otimes |z_+\rangle\langle z_+| \otimes |z_+\rangle\langle z_+|$ respectively, where $|z_+\rangle = (1,0)^T$ and $|x_+\rangle = (1,1)^T/\sqrt{2}$.

By inferring the approximate observables $\mathcal{A}$ and $\mathcal{B}$ we verify that the separable measurement saturates the uncertainty relation whereas the two-copy measurement violates it. As $\mathcal{A}$ and $\mathcal{B}$ commute, the measurement of one does not disturb any subsequent measure of the other. Hence, our collective measurement violating the LW uncertainty relation can be mapped to a violation of error-disturbance type uncertainty relations [32]. We note here that, for the two-copy measurement, we scale the errors ϵ_A and ϵ_B by a factor of two, because they are effectively estimating the optimal operator twice. This rescaling by a factor of two is the same rescaling as for the two-copy CFI. This is necessary, otherwise the same measurement repeated side by side on independent copies of the system would give a measurement error for the complete system which is at least a factor of two greater than the measurement error for the individual system.

It has been known for some time that the original formulation of the uncertainty principle was not a tight bound, and indeed Heisenberg's uncertainty principle has been violated experimentally [36–38]. However, prior to this work, it was not demonstrated, theoretically or experimentally, that the universally valid uncertainty relations which succeeded Heisenberg's uncertainty principle, could be violated. The metrological bound derived by Lu and Wang [30] built on several previous uncertainty relations [32–35]. There is nothing incorrect in any of these works, however they all rely on a particular assumption from Ozawa's paper. In Ref. [33] Ozawa states "We assume that any joint measurements are carried out on single systems". So it is built into the definition of all of these uncertainty relations that only separable measurements are considered. As collective measurements offer no advantage over separable measurements for pure states, we can expect the above uncertainty relations to hold for pure states.

Supplementary Note 11 . Adjusting Lu and Wang's bound to allow for collective measurements

Eq. (19) of the supplemental material of Lu and Wang's paper, Ref. [30], reads

$$R_{jj}\mathcal{F}_{kk} + R_{kk}\mathcal{F}_{jj} + 2\sqrt{\mathcal{F}_{jj}\mathcal{F}_{kk} - D_{jk}^2}\sqrt{R_{jj}R_{kk}} \geq D_{jk}^2 \ , \tag{67}$$

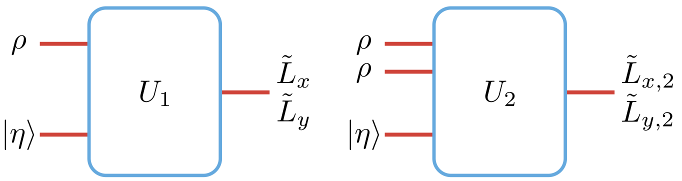

FIG. S7. **Schematic for estimating SLD operators.** As the SLD operators for this problem do not commute, it is necessary to measure an approximate version of these operators on an extended Hilbert space. ρ is the input state and $|\eta\rangle$ is any ancilla state. The unitary matrices to be implemented are those that satisfy Eq. (62). The two-copy measurement is able to estimate the two-copy SLD operators better than what is allowed by the uncertainty relation, Eq. (56), after accounting for a factor of two rescaling. $\tilde{L}_{x(y)}$ and $\tilde{L}_{x(y),2}$ are the approximate observables measured in the single- and two-copy schemes respectively.

where we have replaced C^2_{jk} with D^2_{jk}, which strengthens the inequality. This bound holds when the regret is the difference between the SLD Fisher information and a separable measurement precision, $R_{jj} = \mathcal{F}_{jj} - F^{\text{sep}}_{jj}$. However, in reality when considering collective measurement precisions the regret can be reduced. By examining how much the regret can decrease when allowing for collective measurements, the LW uncertainty relation can be altered to accounts for collective measurements. We denote the CFI for the optimal collective measurement, i.e. a collective measurement on infinitely many copies of the probe state, as F^{col}_{jj}. Then the regret R_{jj} can be reduced a factor S_{jj} where

$$S_{jj} = \frac{\mathcal{F}_{jj} - F^{\text{col}}_{jj}}{\mathcal{F}_{jj} - F^{\text{sep}}_{jj}} \leq 1 \; . \tag{68}$$

We can therefore modify Eq. (67) in the following way to account for collective measurements

$$R_{jj}\mathcal{F}_{kk} + R_{kk}\mathcal{F}_{jj} + 2\sqrt{\mathcal{F}_{jj}\mathcal{F}_{kk} - D^2_{jk}}\sqrt{R_{jj}R_{kk}} \geq D^2_{jk} \times \min(S_{jj}, S_{kk}) \; . \tag{69}$$

Unfortunately, S_{jj} is not easily computed as there is no known way to find F^{col}_{jj}. Nevertheless, there are still situations where Eq. (69) will be useful. For symmetric problems we have that $F^{\text{col}}_{jj} = F^{\text{col}}_{kk} = 2/\mathcal{H}$. As the Holevo bound can be computed efficiently, $\min(S_{jj}, S_{kk})$ can be computed efficiently in this case.

Supplementary Note 12 . Probability simplex

In order to extract information about θ, varying θ must change the probability of the measurement outcomes obtained. The different possible probability distributions form a space known as a probability simplex [39]. In Fig S8 we plot the the probability simplex generated by varying θ_x and θ_y in the region 0 to 2π, as a geometrical interpretation of our two-copy measurement. As we can only plot a 3D simplex we combine two of the outcome probabilities into one axis. The three different axes of our simplex are p_1, p_2 and $p_3 + p_4$, where p_i is the probability of obtaining the ith measurement outcome from Eq. (20). The probability simplex is shown for different values of ϵ. When ϵ increases the area occupied by the simplex decreases, meaning neighbouring states are harder to distinguish. As $\epsilon \to 1$ the simplex shrinks to the point where all four measurement outcomes are equally likely for all values of θ. At this point it is impossible to discern any information about θ and so the variance goes to infinity.

Supplementary References

[1] Holevo, A. S. Statistical decision theory for quantum systems. *J. Multivar. Anal.* **3**, 337–394 (1973).

[2] Holevo, A. S. *Probabilistic and statistical aspects of quantum theory*, vol. 1 (Springer Science & Business Media, 2011).

[3] Nagaoka, H. A new approach to cramér-rao bounds for quantum state estimation. In *Asymptotic Theory Of Quantum Statistical Inference: Selected Papers*, 100–112 (2005). Originally published as IEICE Technical Report, 89, 228, IT 89-42, 9-14, (1989).

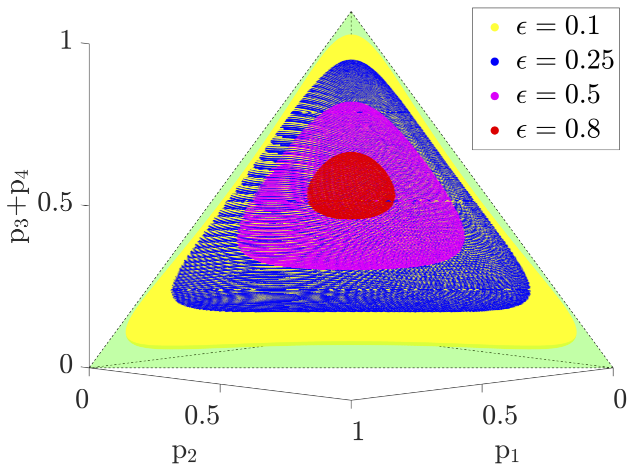

FIG. S8. **Probability simplex for the problem being considered.** Varying θ_x and θ_y generates a space known as a probability simplex. The green tetrahedron is the set of allowed probabilities. As there are only four measurement outcomes, the probability simplexes generated are restricted to lie on the surface of the tetrahedron. The probability simplexes decrease in size as ϵ increases which accounts for the increasing variance.

[4] Nagaoka, H. A generalization of the simultaneous diagonalization of hermitian matrices and its relation to quantum estimation theory. In *Asymptotic Theory Of Quantum Statistical Inference: Selected Papers*, 133–149 (World Scientific, 2005). Originally published as Trans. Jap. Soc. Indust. Appl. Math., 1, 43-56, (1991) in Japanese. Translated to English by Y.Tsuda.

[5] Albarelli, F., Friel, J. F. & Datta, A. Evaluating the holevo cramér-rao bound for multiparameter quantum metrology. *Phys. Rev. Lett.* **123**, 200503 (2019).

[6] Conlon, L. O., Suzuki, J., Lam, P. K. & Assad, S. M. Efficient computation of the nagaoka–hayashi bound for multiparameter estimation with separable measurements. *npj Quantum Inf.* **7**, 1–8 (2021).

[7] Neumark, M. On spectral functions of a symmetric operator. *Izvestiya Rossiiskoi Akademii Nauk. Seriya Matematicheskaya* **7**, 285–296 (1943).

[8] Conlon, L. Github repository. `https://github.com/LorcanConlon/Approaching-optimal-entangling-collective-measurements` (2013).

[9] Vatan, F. & Williams, C. Optimal quantum circuits for general two-qubit gates. *Phys. Rev. A* **69**, 032315 (2004).

[10] Vatan, F. & Williams, C. P. Realization of a general three-qubit quantum gate. *arXiv preprint quant-ph/0401178* (2004).

[11] Roccia, E. *et al.* Entangling measurements for multiparameter estimation with two qubits. *Quantum Sci. Tech.* **3**, 01LT01 (2017).

[12] Parniak, M. *et al.* Beating the rayleigh limit using two-photon interference. *Phys. Rev. Lett.* **121**, 250503 (2018).

[13] Hou, Z. *et al.* Deterministic realization of collective measurements via photonic quantum walks. *Nat. Commun.* **9**, 1–7 (2018).

[14] Wu, K.-D. *et al.* Experimentally reducing the quantum measurement back action in work distributions by a collective measurement. *Sci. Adv.* **5**, eaav4944 (2019).

[15] Yuan, Y. *et al.* Direct estimation of quantum coherence by collective measurements. *npj Quantum Inf.* **6**, 1–5 (2020).

[16] Wang, X.-L. *et al.* 18-qubit entanglement with six photons' three degrees of freedom. *Phys. Rev. Lett.* **120**, 260502 (2018).

[17] Kurzyński, P. & Wójcik, A. Quantum walk as a generalized measuring device. *Phys. Rev. Lett.* **110**, 200404 (2013).

[18] Bian, Z. *et al.* Realization of single-qubit positive-operator-valued measurement via a one-dimensional photonic quantum walk. *Phys. Rev. Lett.* **114**, 203602 (2015).

[19] Zhao, Y.-y. *et al.* Experimental realization of generalized qubit measurements based on quantum walks. *Phys. Rev. A* **91**, 042101 (2015).

[20] Li, Z., Zhang, H. & Zhu, H. Implementation of generalized measurements on a qudit via quantum walks. *Phys. Rev. A* **99**, 062342 (2019).

[21] Wang, X.-L. *et al.* Experimental ten-photon entanglement. *Phys. Rev. Lett.* **117**, 210502 (2016).

[22] Zhong, H.-S. *et al.* 12-photon entanglement and scalable scattershot boson sampling with optimal entangled-photon pairs from parametric down-conversion. *Phys. Rev. Lett.* **121**, 250505 (2018).

[23] Czarnik, P., Arrasmith, A., Coles, P. J. & Cincio, L. Error mitigation with clifford quantum-circuit data. *Quantum* **5**, 592 (2021).

[24] Temme, K., Bravyi, S. & Gambetta, J. M. Error mitigation for short-depth quantum circuits. *Phys. Rev. Lett.* **119**, 180509 (2017).

[25] Kandala, A. *et al.* Error mitigation extends the computational reach of a noisy quantum processor. *Nature* **567**, 491–495 (2019).

[26] Li, Y. & Benjamin, S. C. Efficient variational quantum simulator incorporating active error minimization. *Phys. Rev. X* **7**, 021050 (2017).

[27] Maciejewski, F. B., Zimborás, Z. & Oszmaniec, M. Mitigation of readout noise in near-term quantum devices by classical post-processing based on detector tomography. *Quantum* **4**, 257 (2020).

[28] Mooney, G. J., White, G. A., Hill, C. D. & Hollenberg, L. C. Generation and verification of 27-qubit greenberger-horne-zeilinger states in a superconducting quantum computer. *J. Phys. Commun.* **5**, 095004 (2021).

[29] Yamamoto, K., Endo, S., Hakoshima, H., Matsuzaki, Y. & Tokunaga, Y. Error-mitigated quantum metrology via virtual purification. *arXiv preprint arXiv:2112.01850* (2022).

[30] Lu, X.-M. & Wang, X. Incorporating heisenberg's uncertainty principle into quantum multiparameter estimation. *Phys. Rev. Lett.* **126**, 120503 (2021).

[31] Fujiwara, A. & Nagaoka, H. An estimation theoretical characterization of coherent states. *J. Math. Phys.* **40**, 4227–4239 (1999).

[32] Ozawa, M. Universally valid reformulation of the heisenberg uncertainty principle on noise and disturbance in measurement. *Phys. Rev. A* **67**, 042105 (2003).

[33] Ozawa, M. Uncertainty relations for joint measurements of noncommuting observables. *Phys. Lett. A* **320**, 367–374 (2004).

[34] Branciard, C. Error-tradeoff and error-disturbance relations for incompatible quantum measurements. *Proc. Natl. Acad. Sci.* **110**, 6742–6747 (2013).

[35] Ozawa, M. Error-disturbance relations in mixed states. *arXiv preprint arXiv:1404.3388* (2014).

[36] Erhart, J. *et al.* Experimental demonstration of a universally valid error–disturbance uncertainty relation in spin measurements. *Nat. Phys.* **8**, 185–189 (2012).

[37] Rozema, L. A. *et al.* Violation of heisenberg's measurement-disturbance relationship by weak measurements. *Phys. Rev. Lett.* **109**, 100404 (2012).

[38] Demirel, B., Sponar, S., Sulyok, G., Ozawa, M. & Hasegawa, Y. Experimental test of residual error-disturbance uncertainty relations for mixed spin-1/2 states. *Phys. Rev. Lett.* **117**, 140402 (2016).

[39] Sidhu, J. S. & Kok, P. Geometric perspective on quantum parameter estimation. *AVS Quantum Sci.* **2**, 014701 (2020).
